# Comparison of Contemporary Risk Scores in All Groups of Pulmonary Hypertension: A Pulmonary Vascular Research Institute GoDeep Meta-Registry Analysis

**DOI:** 10.1016/j.chest.2024.03.018

**Published:** 2024-03-19

**Authors:** Athiththan Yogeswaran, Henning Gall, Meike Fünderich, Martin R. Wilkins, Luke Howard, David G. Kiely, Allan Lawrie, Paul M. Hassoun, Yuriy Sirenklo, Olena Torbas, Andrew J. Sweatt, Roham T. Zamanian, Paul G. Williams, Marlize Frauendorf, Alexandra Arvanitaki, George Giannakoulas, Khaled Saleh, Hani Sabbour, Hector R. Cajigas, Robert Frantz, Imad Al Ghouleh, Stephen Y. Chan, Evan Brittain, Jeffrey S. Annis, Antonella Pepe, Stefano Ghio, Stylianos Orfanos, Anastasia Anthi, Raphael W. Majeed, Jochen Wilhelm, Hossein Ardeschir Ghofrani, Manuel J. Richter, Friedrich Grimminger, Sandeep Sahay, Khodr Tello, Werner Seeger, Pulmonary Vascular Research Institute GoDeep Consortium, Tobiah Antoine, Achim Backofen, John Cannon, Victoria Damonte, Diego Echazarreta, Christina Eichstaedt, Jean Elwing, Kai Förster, Ekkehard Gruenig, Anne Hilgendorff, Arun Jose, Ernesto Junaeda, Philipp Krieb, Kurt Marquardt, Karen Osborn, Johanna Pepke-Zaba, Ioan Tilea, Andreea Varga

**Affiliations:** r Department of Internal Medicine, Universities of Giessen and Marburg Lung Center (UGMLC), Member of the German Center for Lung Research (DZL), Giessen, Germany; s Thoraxklinik Heidelberg, Heidelberg, Germany; t Ludwig-Maximillian-University, Munich, Germany; u Royal Papworth Hospital, Cambridge, England; v Pulmonary Vascular Research Institute, Canterbury, England; w University of Cordoba, Cordoba, Argentina; x Universidad Nacional de La Plata, La Plata, Argentina; y University of Cincinnati, Cincinnati, OH; z George Emil Palade University of Medicine, Pharmacy, Science and Technology, Târgu Mures, Romania; a Department of Internal Medicine, Universities of Giessen and Marburg Lung Center, Member of the German Center for Lung Research, Giessen, Germany; b Institute for Lung Health, Cardio-Pulmonary Institute (CPI), Giessen, Germany; c Institute of Medical Informatics, RWTH Aachen University, Aachen, Germany; d National Heart and Lung Institute, Imperial College London, London; e Sheffield Pulmonary Vascular Disease Unit, Royal Hallamshire Hospital, University of Sheffield and National Institute for Health and Care Research Sheffield Biomedical Research Centre, Sheffield, England; f Division of Pulmonary and Critical Care Medicine, Department of Medicine, Johns Hopkins University School of Medicine, Baltimore, MD; g Division of Pulmonary, Allergy, and Critical Care and the Vera Moulton Wall Center for Pulmonary Vascular Disease, Stanford University, Palo Alto, CA; h Division of Pulmonary and Critical Care Medicine, Mayo Clinic, Rochester, MN; i University of Pittsburgh, Pittsburgh, PA; j Vanderbilt University Medical Center, Nashville, TN; k Houston Methodist Hospital, Houston, TX; l National Scientific Center M.D. Strazhesko Institute of Cardiology, Clinical and Regenerative Medicine, The National Academy of Medical Sciences of Ukraine, Kyiv, Ukraine; m Milpark Netcare Hospital, Johannesburg, South Africa; n First Department of Cardiology, Aristotle University of Thessaloniki, Thessaloniki, Greece; o Evangelismos Hospital Athens, Athens, Greece; p Cleveland Clinic Abu Dhabi, Abu Dhabi, United Arab Emirates; q Fondazione IRCCS Policlinico S. Matteo, Pavia, Italy

**Keywords:** multicenter, predictive power, pulmonary hypertension, PVRI GoDeep meta-registry, risk stratification

## Abstract

**Background:**

Pulmonary hypertension (PH) is a heterogeneous disease with a poor prognosis. Accurate risk stratification is essential for guiding treatment decisions in pulmonary arterial hypertension (PAH). Although various risk models have been developed for PAH, their comparative prognostic potential requires further exploration. Additionally, the applicability of risk scores in PH groups beyond group 1 remains to be investigated.

**Research Question:**

Are risk scores originally developed for PAH predictive in PH groups 1 through 4?

**Study Design and Methods:**

We conducted a comprehensive analysis of outcomes among patients with incident PH enrolled in the multicenter worldwide Pulmonary Vascular Research Institute GoDeep meta-registry. Analyses were performed across PH groups 1 through 4 and further subgroups to evaluate the predictive value of PAH risk scores, including the Registry to Evaluate Early and Long-Term PAH Disease Mangement (REVEAL) Lite 2, REVEAL 2.0, European Society of Cardiology/European Respiratory Society 2022, Comparative, Prospective Registry of Newly Initiated Therapies for Pulmonary Hypertension (COMPERA) 3-strata, and COMPERA 4-strata.

**Results:**

Eight thousand five hundred sixty-five patients were included in the study, of whom 3,537 patients were assigned to group 1 PH, whereas 1,807 patients, 1,635 patients, and 1,586 patients were assigned to group 2 PH, group 3 PH, and group 4 PH, respectively. Pulmonary hemodynamics were impaired with median mean pulmonary arterial pressure of 42 mm Hg (interquartile range, 33-52 mm Hg) and pulmonary vascular resistance of 7 Wood units (WU) (interquartile range, 4-11 WU). All risk scores were prognostic in the entire PH population and in each of the PH groups 1 through 4. The REVEAL scores, when used as continuous prediction models, demonstrated the highest statistical prognostic power and granularity; the COMPERA 4-strata risk score provided subdifferentiation of the intermediate-risk group. Similar results were obtained when separately analyzing various subgroups (PH subgroups 1.1, 1.4.1, and 1.4.4; PH subgroups 3.1 and 3.2; group 2 with isolated postcapillary PH vs combined precapillary and postcapillary PH; patients of all groups with concomitant cardiac comorbidities; and severe [> 5 WU] vs nonsevere PH).

**Interpretation:**

This comprehensive study with real-world data from 15 PH centers showed that PAH-designed risk scores possess predictive power in a large PH cohort, whether considered as common to the group or calculated separately for each PH group (1-4) and various subgroups.

**Trial Registry:**

ClinicalTrials.gov; No.: NCT05329714; URL: www.clinicaltrials.gov

FOR EDITORIAL COMMENT, SEE PAGE 420

**Take-home Points**

**Study Question:** Do risk scores originally developed for pulmonary arterial hypertension (PAH; group 1 pulmonary hypertension [PH]) have predictive power in patients with non-PAH PH?

**Results:** Three-strata and four-strata risk scores predicted survival in all PH groups 1 through 4, with the Comparative, Prospective Registry of Newly Initiated Therapies for Pulmonary Hypertension (COMPERA) four-strata risk score effectively distinguishing patients with high-risk and low-risk disease within the intermediate-risk group, whereas the Registry to Evaluate Early and Long-Term PAH Disease Management (REVEAL) scores, used as a continuous scoring system, showed the highest statistical prognostic power and granularity in this global multicenter study including 8,565 patients with incident and treatment-naïve disease.

**Interpretation:** PAH-designed three-strata, four-strata, or continuous data risk scores possess predictive power in a large cohort of patients with PH, whether considered as a common group or calculated separately for each PH group (1-4) as well as various subgroups.

Pulmonary hypertension (PH) is a multifaceted and heterogeneous disease with classification into five distinct groups: group 1, pulmonary arterial hypertension (PAH); group 2, PH associated with left heart disease; group 3, PH associated with lung disease, hypoxia, or both; group 4, PH associated with pulmonary artery obstruction (CTEPH); and group 5, PH with an unclear or multifactorial cause, or both.^1^ It is noteworthy that the survival of all patients with PH is compromised substantially when compared with that of individuals without PH.^2^, ^3^, ^4^, ^5^ Particularly in PAH, risk stratification plays a pivotal role because it guides essential treatment decisions, including the consideration of parenteral prostacyclin therapy for high-risk patients.^1^ Among the critical determinants of symptoms and prognosis in patients with PH, right ventricular function stands out.^6^ It is well known that right ventricular function is compromised across all PH groups, making it pertinent to evaluate the applicability of risk stratification originally designed for PAH to PH groups 2 through 4, a subject that has only received limited attention in previous studies.^7^, ^8^, ^9^, ^10^, ^11^

In Europe, a comprehensive risk score, initially introduced in the 2015 European Society of Cardiology (ESC) and European Respiratory Society (ERS) guidelines on PH, was designed to assess risk in patients with PAH and recently was updated in the latest guidelines.^1^^,^^12^ However, in the absence of specific recommendations for calculating overall risk, various methods have emerged, including calculating the mean with rounding to the nearest integer or simply tallying low-risk parameters using a truncated version of the risk score.^10^^,^^13^, ^14^, ^15^ In the United States, the Registry to Evaluate Early and Long-Term PAH Disease Management (REVEAL) 2.0 risk score and the REVEAL Lite 2 risk score are preferred tools for assessing mortality risk in patients with PAH.^16^ Both risk assessment tools categorize patients into three risk groups: low risk, intermediate risk, and high risk.^1^^,^^12^^,^^16^ A key distinction between the REVEAL and the ESC and ERS approaches lies in the inclusion of demographics, such as sex and age, as well as PAH subtype analysis in the REVEAL 2.0 score, but also in the possibility to use the REVEAL scores as a continuous (ordinal) scoring system.^16^, ^17^, ^18^, ^19^, ^20^

In addition to the three-strata risk models mentioned so far, four-strata risk models have been developed to provide a more comprehensive characterization of patients during follow-up.^1^^,^^14^^,^^15^ Although the current ESC and ERS guidelines recommend using a three-strata risk approach at the time of diagnosis and a four-strata risk approach during follow-up, the added benefits of using four-strata risk scores during baseline evaluation remain uncertain.^1^ Notable, the recommended four-strata risk score does not encompass pulmonary hemodynamics, whereas the ESC and ERS three-strata risk score incorporates pulmonary hemodynamics as well as imaging and cardiopulmonary exercise testing findings. The extent to which including hemodynamic measurements adds prognostic value remains to be determined.

Although certain parameters are common to all risk assessment scores, such as 6-min walking distance (6MWD) and World Health Organization (WHO) functional class, major components differ between the various scores (e-Table 1). Furthermore, no consensus exists regarding whether the PAH-designed risk scores can be extended usefully to PH groups 2 through 4. This study used the large Pulmonary Vascular Research Institute (PVRI) GoDeep metaregistry^21^ to compare the predictive power of both three-strata (including REVEAL scores, additionally allowing a continuous scoring approach) and four-strata risk scores in a large multicenter cohort. Moreover, it aimed to investigate whether these risk scores are equally applicable to patients assigned to groups 2 through 4 PH.

## Study Design and Methods

### Study Population

All patients enrolled in the PVRI GoDeep meta-registry with right heart catheter-confirmed PH diagnosis made by the participating PH expert center based on the PH World Symposium definition of PH, age at diagnosis of ≥ 18 years, and without any data discrepancies were included in this study.^21^ The time range for baseline data was set at −3 to +3 months around the time of reported initial diagnosis. If multiple data points were available for the same variable, the data point closest to the diagnosis date was selected. The current analysis included all centers from which sufficiently granular data for comparative risk sore analysis could be entered into the study, namely the centers in Giessen (2,198 patients), London (2,143 patients), Sheffield (2,023 patients), Baltimore (632 patients), Kyiv (380 patients), Stanford (342 patients), Johannesburg (220 patients), Thessaloniki (156 patients), Abu Dhabi (117 patients), Rochester (109 patients), Houston (87 patients), Pittsburgh (76 patients), Nashville (46 patients), Pavia (28 patients), and Athens (8 patients). The University of Giessen University Hospital Ethics Committee and the responsible local ethic committees approved the PVRI GoDeep central data repository, listed at ClinicalTrials.gov (Identifier: NCT05329714).

### Risk Assessment Models

We included the REVEAL Lite 2 risk score, the REVEAL 2.0 risk score, the ESC and ERS 2022 risk score, and the Comparative, Prospective Registry of Newly Initiated Therapies for Pulmonary Hypertension (COMPERA) three-strata and four-strata risk scores (e-Table 1).

#### REVEAL Lite 2 and REVEAL 2.0 Risk Scores

As described by Benza and coworkers,^20^ the REVEAL 2.0 score was calculated using the following variables: WHO group 1 subgroup, demographics, estimated glomerular filtration rate, WHO functional class, vital signs (systolic BP and heart rate), 6MWD, B-type natriuretic peptide (BNP) level, presence of pericardial effusion, lung function test results (ie, diffusion capacity of the lungs for carbon monoxide), and right heart catheterization data (ie, mean right artery pressure and pulmonary vascular resistance (PVR) at the time of diagnosis). The REVEAL scores were used as a continuous scoring system, unless otherwise noted.^20^ Missing values were substituted by a score of zero.^20^ Similarly, REVEAL Lite 2.0 risk was calculated by incorporating BNP, 6MWD, WHO functional class, systolic BP, heart rate, and estimated glomerular filtration rate.^20^

#### ESC and ERS 2022 risk score

The Kylhammar approach was used with the new threshold and parameters mentioned in the 2022 ESC and ERS guidelines on PH, including WHO functional class, 6MWD, BNP level, right atrial area, tricuspid annular plane systolic excursion to pulmonary artery systolic pressure ratio, presence of pericardial effusion, peak oxygen uptake (VO_2_), minute ventilation (VE) to carbon dioxide production (VCO_2_) slope, right atrial pressure (RAP), concordance index, stroke volume index, and mixed-venous oxygen saturation (SvO_2_).^1^^,^^10^

#### COMPERA Registry Three-Strata and Four-Strata Risk Score

The COMPERA registry three- and four-strata approaches were performed as described by Hoeper and coworkers^14^ using WHO functional class, 6MWD, and BNP level. In brief, each variable was rated as described previously using numbers between 1 and 3 or between 1 and 4, respectively. The mean then was determined and rounded to the nearest whole number.

The prognostic determinants included in each risk score are detailed in e-Table 1, with missing parameters in the GoDeep registry indicated in italics.

### Data Extraction and Statistical Analyses

Data were analyzed with R software version 4.3.0 (R Foundation for Statistical Computing)^22^ using the package survival version 3.5-3.^23^ The package flextable version 0.9.1 was used to create tables and the package mice version 3.15.0 was used for multiple imputations by multivariate imputation by chained equations.^24^^,^^25^

On October 16, 2023, the data were extracted from the database. Missing values for BNP level were calculated from given N-terminal pro BNP (NT-)proBNP values using the following formula:

$$\log_{e}\text{BNP}=\frac{\log_{e}\text{NTproBNP}\;-0.079}{1.348}$$

Median and interquartile range were used to summarize variables in tables.

Missing data were imputed using mice version 3.15.0.^25^ Reliability of imputation is shown in e-Figure 1. Patients were considered for imputation if they had at least two of the following variables: WHO functional class, 6MWD, and BNP. Missing data were allowed up to 40%, and continuous variables were log transformed before imputation. The following variables were imputed (percent of missing values in parentheses): WHO functional class (4%), body surface area (10%), BMI (13%), height (14%), 6MWD (18%), mean pulmonary arterial pressure (18%), PVR (27%), systolic systemic arterial pressure (28%), pulmonary arterial wedge pressure (36%), BNP (36%), cardiac output (38%), and concordance index (38%). Additionally, the information from following variables without missing values was used for imputation: PH group, sex, age at diagnosis, diagnosis decade, and center.

Kaplan-Meier estimators with log-rank tests as well as univariate and multivariate Cox regression analyses were used to examine the prognostic relevance of parameters. In addition, predictive power was evaluated with Akaike information criteria and C statistic.^26^ Akaike information criteria values were compared with the respective Akaike information criteria values of the ESC and ERS risk score and for the C statistic, bootstrapping was used to determine the statistical significance of the difference between the respective risk score and the ESC and ERS risk score.

## Results

### Baseline Characteristics

**Figure 1:**
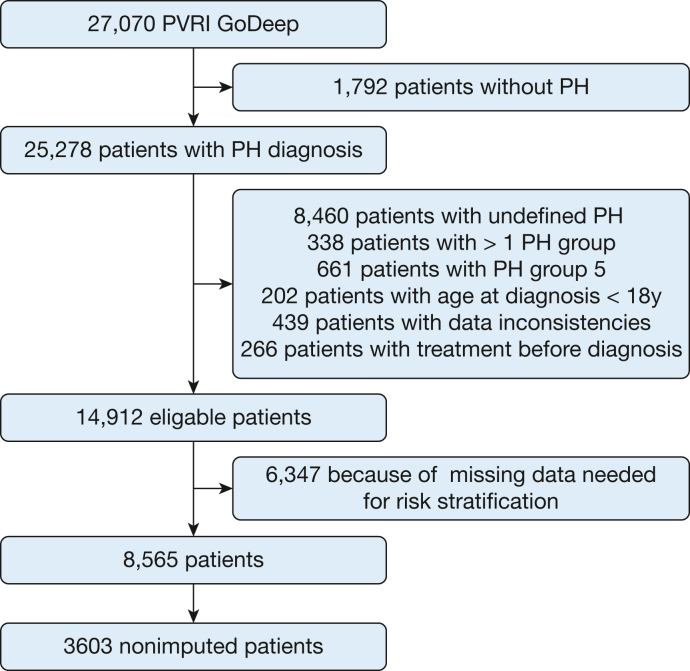
Study flow chart illustrating the sequential steps of the study design. PH = pulmonary hypertension; PVRI = Pulmonary Vascular Research Institute.

As of October 16, 2023, the PVRI GoDeep meta-registry comprised a total of 27,070 patients, including 8,565 patients with incident disease and those who were treatment naïve, enrolled in 15 different PH centers, all of whom met the described criteria for analysis (Fig 1). Among these patients, 3,537 patients (41%) received a diagnosis of group 1 PH, whereas 1,807 patients (21%), 1,635 patients (19%), and 1,586 patients (19%) received a diagnosis of group 2 PH, group 3 PH, and group 4 PH, respectively (Table 1). One hundred one patients with a diagnosis of group 5 PH were excluded from subsequent analyses because of the low patient count and the inherent heterogeneity within this group. The median age of the study population was 65 years (interquartile range, 52-74 years), 39% were male, and the pulmonary hemodynamics were severely impaired, with median pulmonary arterial pressure of 42 mm Hg (interquartile range, 33-52 mm Hg) and median pulmonary vascular resistance of 7 Wood units (WU) (interquartile range, 4-11 WU), as detailed in Table 2. Overall, 1-year, 3-year, and 5-year survival rates were 85%, 64%, and 51%, respectively. Table 3 presents the distribution of the included risk scores. Most patients were categorized as having intermediate risk using the ESC and ERS 2022 risk score and the COMPERA registry three-strata score (81% respectively), whereas the REVEAL 2.0 score showed a more balanced distribution (Table 3).

**Table 1:** Baseline Characteristics of Study Population Stratified by PH Group

Characteristic	PH Group					
	1	2	3	4	Overall	5^a^
No. of patients	3,537	1,807	1,635	1,586	8,565	101
Age at diagnosis, y	57 (43-69)	73 (65-78)	67 (58-73)	66 (52-74)	65 (52-74)	67 (52-73)
Missing data	0 (0)	0 (0)	0 (0)	0 (0)	0 (0)	0 (0)
Sex, male	1,061 (30)	659 (36.5)	836 (51.1)	811 (51.1)	3,367 (39.3)	42 (41.6)
Missing data	0 (0)	0 (0)	0 (0)	0 (0)	0 (0)	0 (0)
WHO functional class						
I	61 (1.72)	41 (2.27)	9 (0.55)	27 (1.7)	138 (1.61)	5 (4.95)
II	573 (16.2)	296 (16.4)	221 (13.5)	226 (14.2)	1,316 (15.4)	7 (16.8)
III	2,380 (67.3)	1,298 (71.8)	1,079 (66)	1,162 (73.3)	5,919 (69.1)	71 (70.3)
IV	523 (14.8)	172 (9.52)	326 (19.9)	171 (10.8)	1,192 (13.9)	8 (7.92)
Missing data	0 (0)	0 (0)	0 (0)	0 (0)	0 (0)	0 (0)
6MWD, m	291 (195-390)	257 (167-350)	243 (174-333)	291 (200-384)	273 (188-367)	244 (152-341)
Missing data	0 (0)	0 (0)	0 (0)	0 (0)	0 (0)	0 (0)
BNP, pg/mL	177 (63.9-422)	199 (91.1-432)	138 (48.5-390)	171 (63.4-426)	175 (65-416)	193 (58-447)
Missing data	0 (0)	0 (0)	0 (0)	0 (0)	0 (0)	0 (0)
mPAP, mm Hg	48 (38-57)	37 (30-45)	37 (29-46)	44 (34-52)	42 (33-52)	43 (34.5-50.5)
Missing data	0 (0)	0 (0)	0 (0)	0 (0)	0 (0)	34 (33.7)
PAWP, mm Hg	10 (7-13)	17 (12-22)	10 (7-14)	10 (8-14)	11 (8-15)	11 (7-14)
Missing data	0 (0)	0 (0)	0 (0)	0 (0)	0 (0)	57 (56.4)
PVR, WU	9.25 (5.59-13.9)	3.52 (2.27-5.74)	5.6 (3.87-9)	7.72 (4.64-11.3)	6.7 (3.87-11)	5.86 (4.32-8.85)
Missing data	0 (0)	0 (0)	0 (0)	0 (0)	0 (0)	50 (49.5)
Concordance index, L/min/m^2^	2.21 (1.77-2.75)	2.54 (2.1-2.97)	2.43 (2-2.88)	2.25 (1.84-2.72)	2.34 (1.89-2.82)	2.73 (2.35-3.1)
Missing data	0 (0)	0 (0)	0 (0)	0 (0)	0 (0)	58 (57.4)

Data are presented as No. (%) or median (interquartile range). 6MWD = 6-min walking distance; BNP = B-type natriuretic peptide; mPAP = mean pulmonary arterial pressure; PAWP = pulmonary arterial wedge pressure; PH = pulmonary hypertension; PVR = pulmonary vascular resistance; WU = Wood unit; WHO = World Health Organization.

a Values for patients with PH in group 5 without imputation.

**Table 2:** Comorbidities Stratified by PH Group

Comorbidity	PH Group				
	1	2	3	4	Overall
No. of patients	3,537	1,807	1,635	1,586	8,565
Obesity	1,212 (34.3)	867 (48)	576 (35.2)	584 (36.8)	3,239 (37.8)
Diabetes mellitus	329 (9.3)	290 (16)	220 (13.5)	106 (6.68)	945 (11)
Coronary heart disease	201 (5.68)	34 (1.88)	49 (3)	13 (0.82)	297 (3.47)
Arterial hypertension	617 (17.4)	538 (29.8)	413 (25.3)	317 (20)	1,885 (22)
Arterial fibrillation	169 (4.78)	490 (27.1)	160 (9.79)	130 (8.2)	949 (11.1)
Renal comorbidities	157 (4.44)	195 (10.8)	163 (9.97)	122 (7.69)	637 (7.44)
Cancer	118 (3.34)	98 (5.42)	141 (8.62)	78 (4.92)	435 (5.08)
Sleep apnea syndrome	259 (7.32)	179 (9.91)	173 (10.6)	90 (5.67)	701 (8.18)
Oxygen treatment	904 (25.6)	200 (11.1)	752 (46)	392 (24.7)	2,248 (26.2)

Data are presented as No. (%) unless otherwise indicated. PH = pulmonary hypertension.

**Table 3:** Risk Score Classification of the Imputed Study Population Stratified by PH Groups 1 Through 4

Variable	PH Group				
	1	2	3	4	Overall
No. of patients	3,537	1,807	1,635	1,586	8,565
REVEAL 2.0					
≤ 6	1,866 (52.8)	1,144 (63.3)	817 (50)	963 (60.7)	4,790 (55.9)
7	433 (12.2)	236 (13.1)	228 (13.9)	207 (13.1)	1,104 (12.9)
8	408 (11.5)	167 (9.24)	201 (12.3)	148 (9.33)	924 (10.8)
9	338 (9.56)	125 (6.92)	155 (9.48)	102 (6.43)	720 (8.41)
10	211 (5.97)	77 (4.26)	104 (6.36)	89 (5.61)	481 (5.62)
11	137 (3.87)	34 (1.88)	75 (4.59)	46 (2.9)	292 (3.41)
12	70 (1.98)	16 (0.885)	33 (2.02)	18 (1.13)	137 (1.6)
≥ 13	74 (2.09)	8 (0.443)	22 (1.35)	13 (0.82)	117 (1.37)
REVEAL Lite 2					
≤ 5	866 (24.5)	348 (19.3)	365 (22.3)	404 (25.5)	1,983 (23.2)
6	484 (13.7)	229 (12.7)	207 (12.7)	197 (12.4)	1,117 (13)
7	603 (17)	370 (20.5)	286 (17.5)	315 (19.9)	1,574 (18.4)
8	647 (18.3)	395 (21.9)	314 (19.2)	294 (18.5)	1,650 (19.3)
9	513 (14.5)	304 (16.8)	250 (15.3)	213 (13.4)	1,280 (14.9)
10	271 (7.66)	127 (7.03)	135 (8.26)	119 (7.5)	652 (7.61)
11	118 (3.34)	29 (1.6)	63 (3.85)	37 (2.33)	247 (2.88)
≥ 12	35 (0.99)	5 (0.277)	15 (0.917)	7 (0.441)	62 (0.724)
ESC and ERS 2022					
Low	220 (6.22)	93 (5.15)	102 (6.24)	89 (5.61)	504 (5.88)
Intermediate	2,790 (78.9)	1,550 (85.8)	1,338 (81.8)	1,271 (80.1)	6,949 (81.1)
High	527 (14.9)	164 (9.08)	195 (11.9)	226 (14.2)	1,112 (13)
COMPERA 3-strata					
Low	439 (12.4)	132 (7.3)	121 (7.4)	173 (10.9)	865 (10.1)
Intermediate	2,794 (79)	1,543 (85.4)	1,337 (81.8)	1,292 (81.5)	6,966 (81.3)
High	304 (8.59)	132 (7.3)	177 (10.8)	121 (7.63)	734 (8.57)
COMPERA 4-strata					
Low	283 (8)	87 (4.81)	74 (4.53)	114 (7.19)	558 (6.51)
Intermediate to low	1,163 (32.9)	527 (29.2)	499 (30.5)	525 (33.1)	2,714 (31.7)
Intermediate to high	1,830 (51.7)	1,082 (59.9)	922 (56.4)	841 (53)	4,675 (54.6)
High	261 (7.38)	111 (6.14)	140 (8.56)	106 (6.68)	618 (7.22)

Data are presented as No. (%) unless otherwise indicated. Only patients with available 6-min walking distance, B-type natriuretic peptide, and World Health Organization functional class were included. COMPERA = Comparative, Prospective Registry of Newly Initiated Therapies for Pulmonary Hypertension; ERS = European Respiratory Society; ESC = European Society of Cardiology; PH = pulmonary hypertension; REVEAL = Registry to Evaluate Early and Long-Term PAH Disease Mangement.

### Prognostic Power of Risk Scores in Patients with Incident and Treatment-Naïve PH

**Figure 2:**
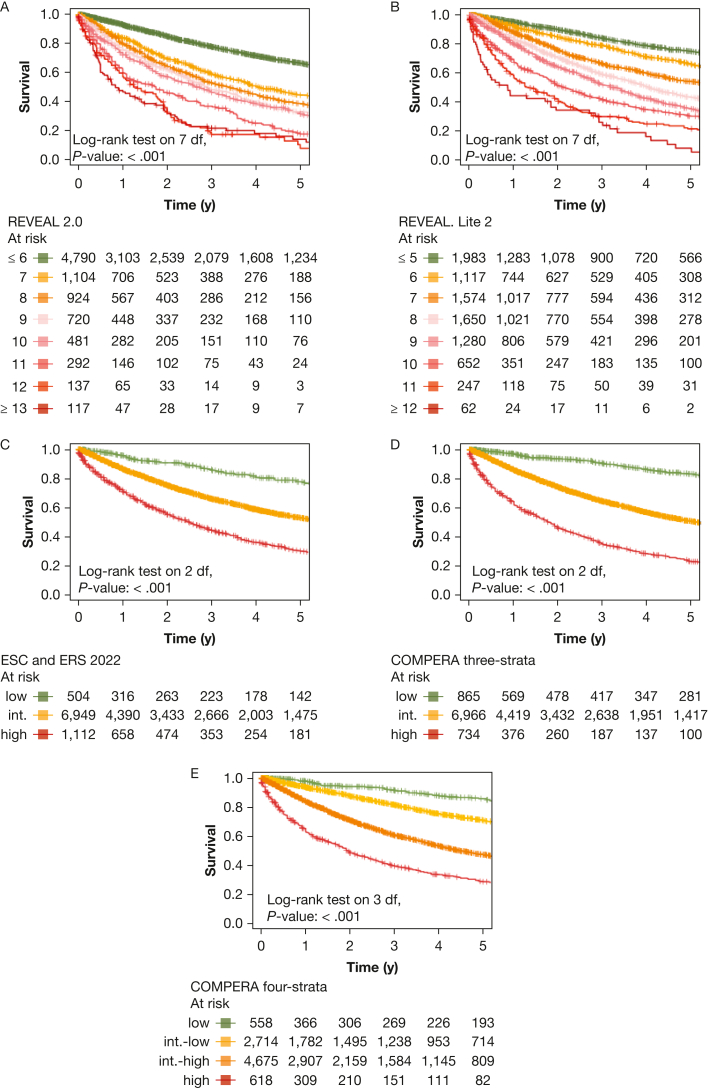
Kaplan-Meier curves of risk scores showing survival of all patients with pulmonary hypertension (PH) stratified by risk score with 95% CIs. A, Kaplan-Meier curve stratified by Registry to Evaluate Early and Long-Term PAH Disease Management (REVEAL) 2.0 risk score. B, Kaplan-Meier curve stratified by REVEAL Lite 2 risk score. C, Kaplan-Meier curve stratified by ERS and ESC 2022 risk score. D, Kaplan-Meier curve stratified by Comparative, Prospective Registry of Newly Initiated Therapies for Pulmonary Hypertension (COMPERA) three-strata risk score. E, Kaplan-Meier curve stratified by COMPERA four-strata risk score. df = degrees of freedom; ERS = European Respiratory Society; ESC = European Society of Cardiology; int. = intermediate.

**Figure 3:**
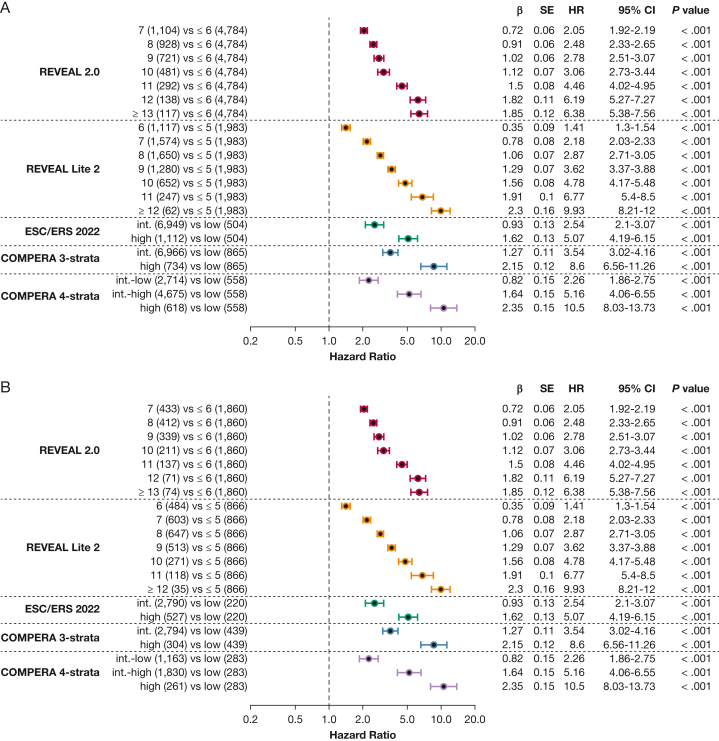

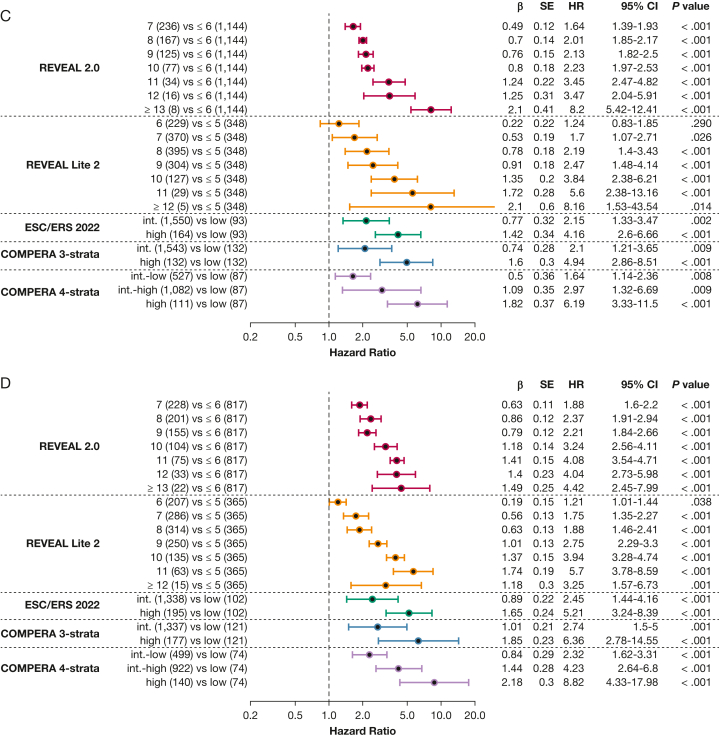

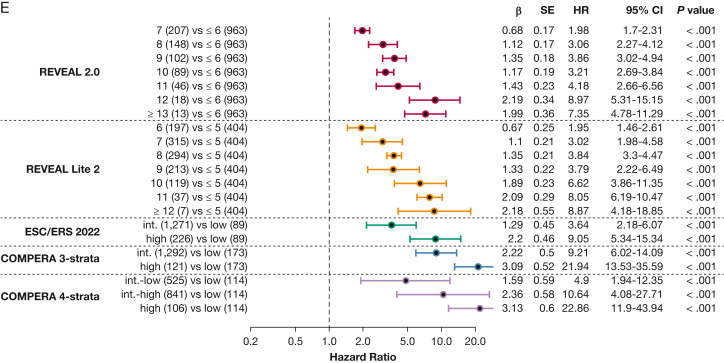
Forest plots showing the included risk scores. A, Forest plot for the overall pulmonary hypertension (PH) group, stratified by risk score. B, Forest plot for PH group 1 stratified by risk score. C, Forest plot for PH group 2 stratified by risk score. D, Forest plot for PH group 3 stratified by risk score. E, Forest plot for PH group 4 stratified by risk score. All plots are in relationship to the low-risk category of the respective risk score. int.-high = intermediate-high; int.-low = intermediate-low.

All risk scores predicted survival in patients with incident PH, as illustrated in Figure 2. The predictive power of all sores is presented in Table 4. Notably, the REVEAL scores significantly outperformed the ESC and ERS 2022 risk score and also compared favorably with the COMPERA registry three-strata risk score. For patients with a REVEAL Lite 2.0 score of ≤ 6, the 1-year, 3-year, and 5-year survival rates were 93%, 78%, and 66%, respectively, in the overall PH population including patients of all PH groups. In contrast, patients with higher scores showed significantly worse prognosis, as depicted in Figure 2A. Univariate Cox regression analysis further confirmed significantly increased hazard ratios for each one-point increase both for REVEAL 2.0 and REVEAL Lite 2, as compared with patients with a total score of ≤ 6 points (Fig 3). The COMPERA registry four-strata risk score successfully discriminated between intermediate-risk to low-risk patients and intermediate-risk to high-risk patients, also significantly outperforming the ESC and ERS 2022 risk score (Table 4). When compared with the low-risk score group, twofold, fivefold, and 11-fold increased hazard ratios were noted for the intermediate-risk to low-risk score patients, intermediate-risk to high-risk score patients, and high-risk score patients (Fig 3). Similarly, significantly increased hazard ratios were noted when comparing patients with intermediate to high risk with patients with intermediate to low risk.

**Table 4:** Predictive Power of the Included Risk Scores

Variable	Reveal 2.0	REVEAL Lite 2	ESC and ERS 2022	COMPERA Three Strata	COMPERA Four Strata
PH overall					
ΔAIC	558	491	0	182	417
Concordance index	0.65^a^	0.66^a^	0.57	0.58^a^	0.63^a^
Group 1					
ΔAIC	294	261	0	64	191
Concordance index	0.68^a^	0.68^a^	0.58	0.59	0.65^a^
Group 2					
ΔAIC	39	37	0	14	32
Concordance index	0.61^a^	0.61^a^	0.56	0.57	0.58
Group 3					
ΔAIC	90	73	0	17	54
Concordance index	0.63^a^	0.63^a^	0.56	0.57	0.60^a^
Group 4					
ΔAIC	60	38	0	16	38
Concordance index	0.66^a^	0.66^a^	0.58	0.59	0.63^a^

Concordance index and the difference of Akaike information criterion estimates between the ESC and ERS 2022 score and the respective risk score of the Cox proportional hazards model based on the entire data set including imputed data are shown. Center and diagnosis decade are included as stratification variables, as is center as a cluster. Values are given for risk scores for overall PH and groups 1 through 4. ΔAIC = difference of Akaike information criterion between the ESC and ERS 2022 score and the respective risk score; COMPERA = Comparative, Prospective Registry of Newly Initiated Therapies for Pulmonary Hypertension; ERS = European Respiratory Society; ESC = European Society of Cardiology; PH = pulmonary hypertension; REVEAL = Registry to Evaluated Early and Long-Term PAH Disease Management.

a *P* < .001 vs ESC and ERS 2022 score.

### Prognostic Power of Risk Scores in Patients With Incident PH Groups 1 Through 4

**Figure 4:**
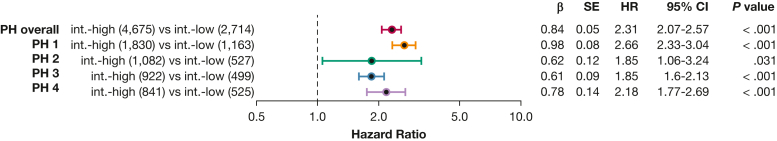
Forest plot showing Comparative, Prospective Registry of Newly Initiated Therapies for Pulmonary Hypertension (COMPERA) four-strata risk score in relationship to the intermediate-risk to low-risk category of the respective risk score. HR = hazard ratio; int.-high = intermediate-high; int.-low = intermediate-low; PH = pulmonary hypertension.

**Figure 5:**
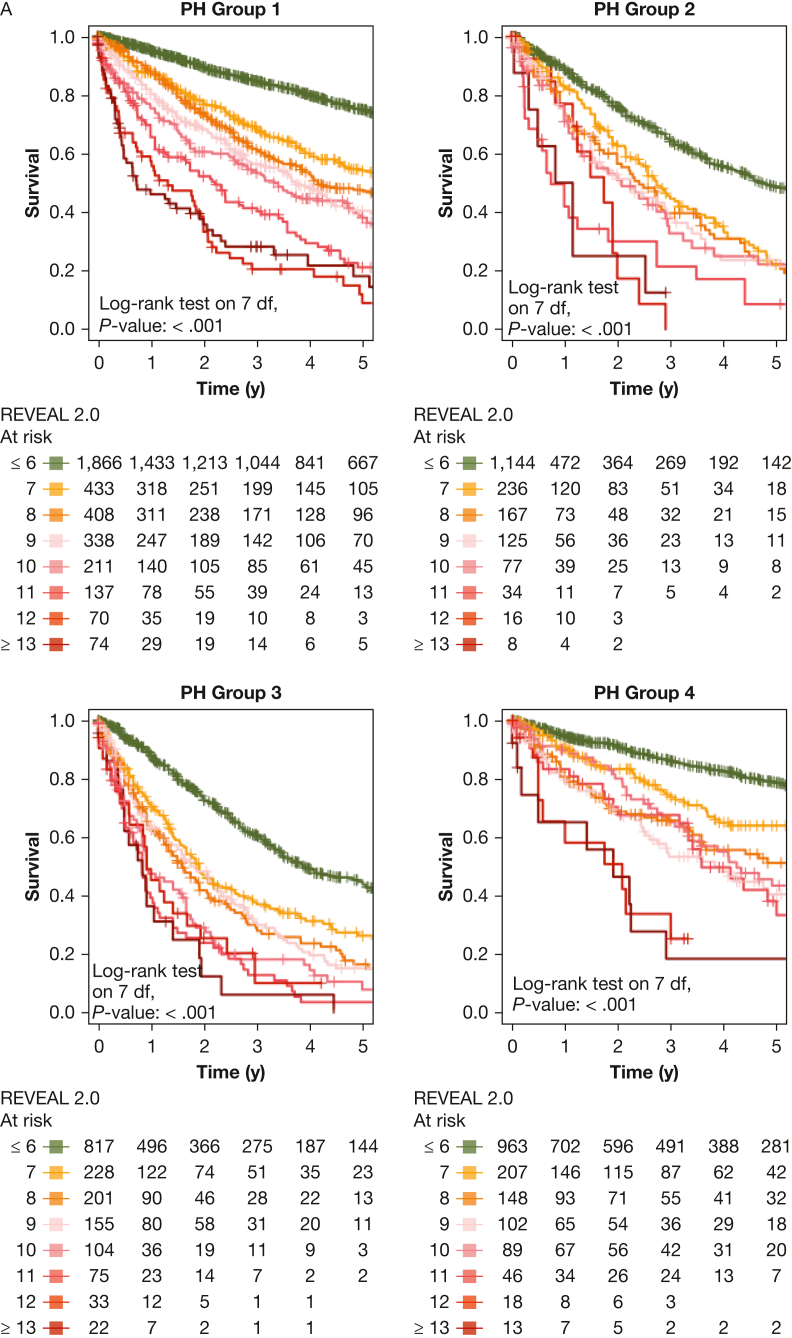

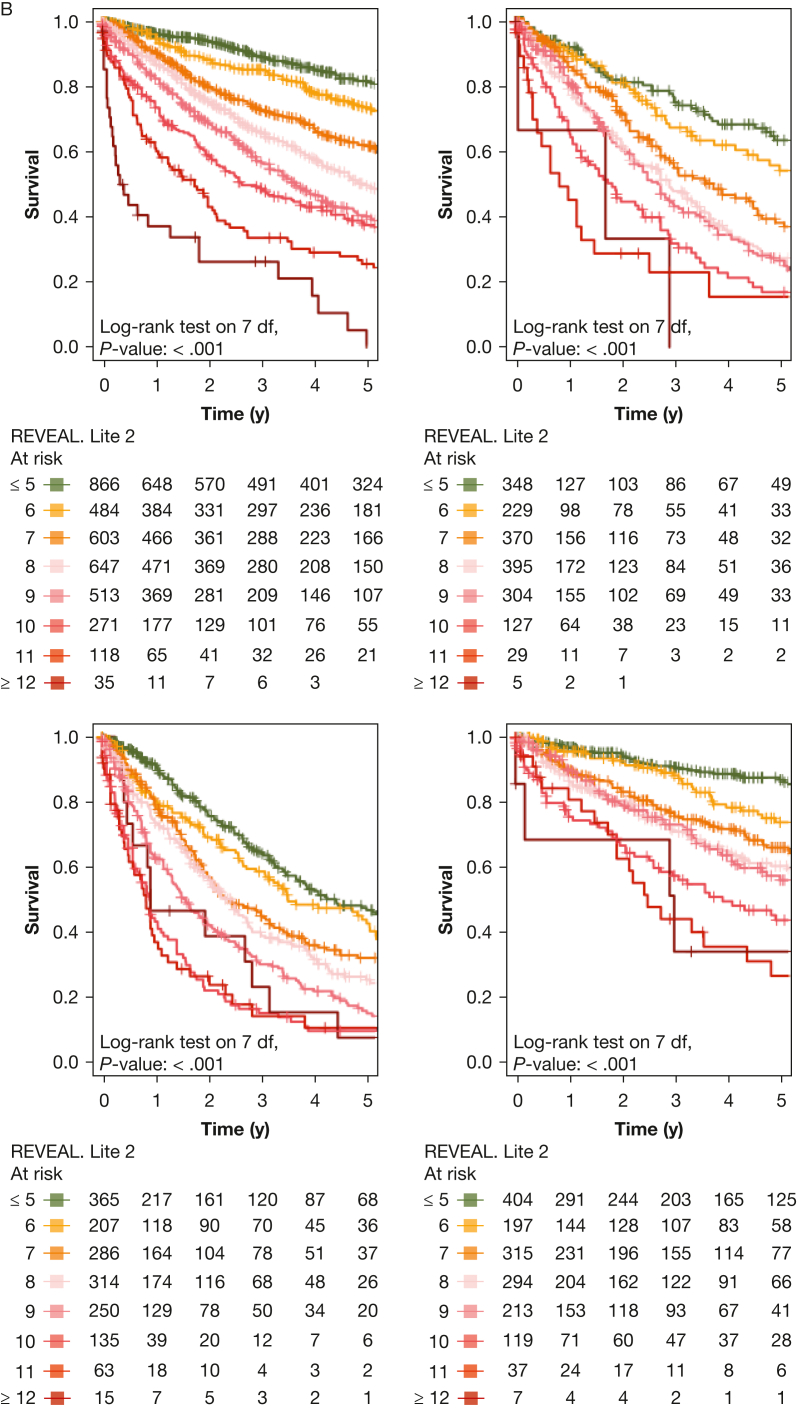

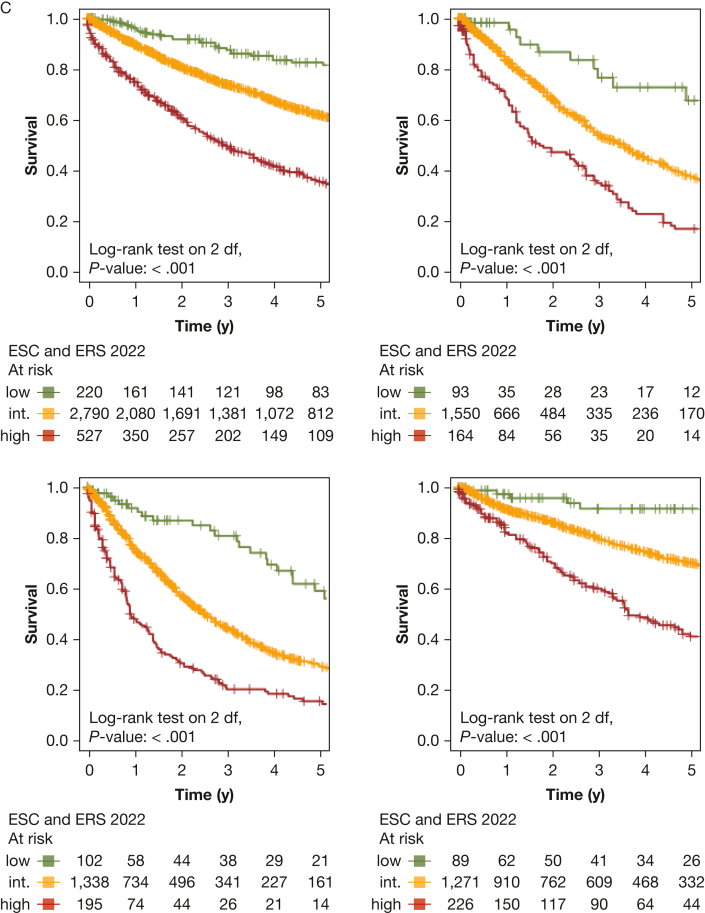

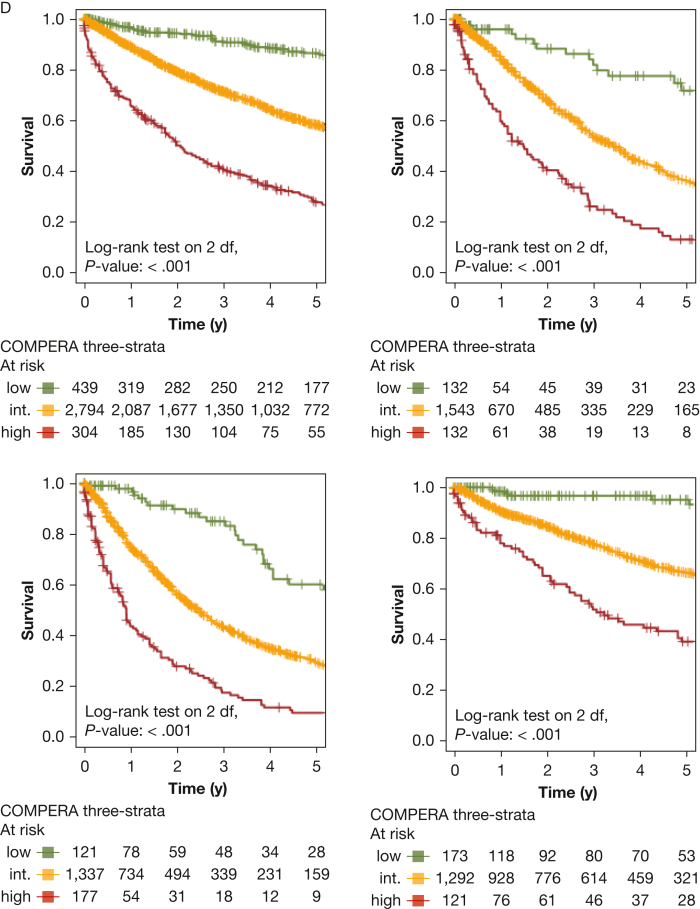

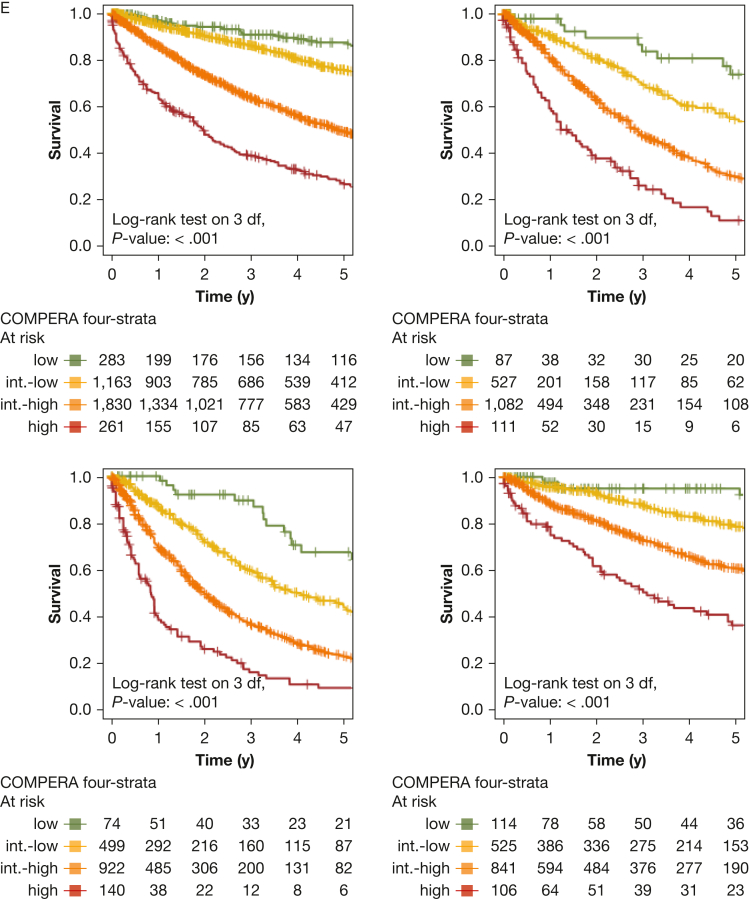
Kaplan-Meier curves showing three-strata and four-strata risk scores for PH groups 1 through 4. Survival rates for each PH group (1-4) are stratified by Registry to Evaluate Early and Long-Term PAH Disease Mangement (REVEAL) 2.0, REVEAL Lite 2, ESC and ERS 2022, Comparative, Prospective Registry of Newly Initiated Therapies for Pulmonary Hypertension (COMPERA) three-strata, and COMPERA four-strata scores, respectively. Kaplan-Meier curves with 95% CIs are shown for patients in each risk score group. Center and diagnosis decade are included as stratification variables, as is center included as cluster. A, Survival rates for REVEAL 2.0. B, Survival rates for REVEAL Lite 2. C, Survival rates for ESC and ERS 2022. D, Survival rates for COMPERA three-strata. E, Survival rates for COMPERA four-strata. ERS = European Respiratory Society; ESC = European Society of Cardiology; int. = intermediate; PH = pulmonary hypertension.

Next, we performed PH group-based analyses, with baseline characteristics shown in Table 1. All included risk scores predicted survival in PH groups 1 through 4 (Figure 4, Figure 5). Kaplan-Meier curves of all scores for all groups are shown in Figure 5. Corresponding to the overall PH group, the ESC and ERS 2022 risk score showed an uneven distribution with strong predominance of the intermediate risk score group in all four PH groups. The COMPERA four-strata risk score was able to discriminate between patients with intermediate to low risk and patients with intermediate to high risk in all PH groups (Figure 4, Figure 5). Again, the C indices of the REVEAL scores, when used as a continuous scoring system, were the highest in each of the individually analyzed PH groups (Table 3).

### Sensitivity Analysis Using Nonimputed Data

To enhance the robustness of our findings, we performed a sensitivity analysis using the subset of patients with incident and treatment-naïve PH without any data imputation (n = 3,603, nonimputed study population). Only patients with complete 6MWD, BNP level, and WHO functional class data sets were included (e-Table 2). Overall, 1-year, 3-year, and 5-year survival rates were 88%, 70%, and 56%, respectively. Baseline characteristics and distributions of the risk scores within the nonimputed cohort are displayed in e-Tables 2-4, respectively. Significantly, outcomes gleaned from this analysis exhibited concurrence with those derived from the analyses incorporating imputation, as delineated in e-Figures 2-5 and e-Table 5. The nonimputed data set again underscores the strong discriminative potency of the continuous REVEAL scores and the efficacy of the four-strata risk score to discriminate between cohorts with intermediate to low risk and intermediate to high risk. This further supports the reliability of the imputation procedure chosen for the current study, also depicted in e-Figure 1.

### Sensitivity Analysis Addressing Specific Subgroups

We conducted additional subgroup analyses to validate our findings across specific patient populations. As depicted in e-Table 6, PAH-designed three-strata and four-strata risk scores were found to possess predictive power also in each of these subgroups, with the particular strong discriminative potency of the continuous REVEAL scores and the efficacy of the four-strata risk score to discriminate between cohorts of intermediate to low risk and intermediate to high risk again being demonstrated. The following subgroups were analyzed: (1) group 1.1, idiopathic pulmonary arterial hypertension; (2) group 1.4.1, connective tissue disease-associated PAH; (3) group 1.4.4, congenital heart disease-associated PAH; (4) group 2, patients with isolated postcapillary PH (ie, PVR ≤ 2 WU); (5) group 2, patients with combined precapillary and postcapillary PH (ie, PVR > 2 WU); (6) group 3.1, PH associated with obstructive lung disease; (7) group 3.2, PH associated with restrictive lung disease; and (8) patients with PAH with cardiac comorbidities (defined as the presence of at least three of the following comorbidities: arterial hypertension, obesity, diabetes, coronary heart disease, and atrial fibrillation). Corresponding results were obtained, when dichotomizing the entire population with PH as well as each of the PH groups for severe vs nonsevere PH (ie, PVR ≥ 5 WU and PVR < 5 WU) (e-Table 6).

## Discussion

This multicenter study comprehensively validated and compared five major existing approaches for risk stratification in PH. It used the largest worldwide meta-registry published to date, the PVRI GoDeep metaregistry.^21^ The major findings are: (1) commonly applied risk stratification schemes are prognostic in patients with PH regardless of clinical subtype; (2) the COMPERA four-strata risk score provides subdifferentiation of the intermediate risk group; (3) including further variables, both modifiable (eg, hemodynamics or renal function) and nonmodifiable (eg, sex and age), adds to the prognostic power in patients with incident and treatment-naïve PH compared with strata models mainly focusing on WHO functional class, BNP level, and 6MWD; and (4) the REVEAL scoring systems, when used as continuous prediction models, possess the highest statistical prognostic power and granularity.

In recent years, the number of studies investigating risk stratification and proposing independent risk parameters for patients with PH has increased rapidly.^8^, ^9^, ^10^, ^11^^,^^13^, ^14^, ^15^^,^^26^, ^27^, ^28^ In parallel, several risk scores have been developed, validated, and established for patients with PAH, including the ESC and ERS risk scores and the REVEAL scores.^1^^,^^12^^,^^18^ The complexity of the risk scores varies from simple noninvasive approaches (eg, the COMPERA registry three-strata and four-strata scores) to more sophisticated invasive approaches (eg, the REVEAL and the ESC and ERS risk scores). Although some parameters are represented in most risk scores (such as 6MWD, WHO functional class, and BNP level), age, sex, PAH subgroup, and renal function are represented only in the REVEAL 2.0 score. Risk scores typically are applied on a national or regional basis.

Determining the most accurate predictive tool is essential because treatment decisions rely on the estimated prognosis, in particular the 1-year prognosis in patients with P(A)H.^1^ The current study extends this approach by exploring a putative predictive power of three-strata and four-strata risk scores for PH groups 2 through 4. To our knowledge, this study is the first to assess the predictive power of the updated version of the ESC and ERS risk score. In particular, BNP level thresholds were changed, leading to more patients being categorized as showing intermediate risk regarding laboratory biomarkers. In addition, imaging gained more importance. When comparing our study with major studies assessing the prognostic power of the 2015 ESC and ERS risk score, our study indicated that the changes lead to substantially more patients being classified as showing intermediate risk. Although still separating patients according to risk, the usefulness of the 2022 ESC and ERS risk score is impaired because > 80% of the patients are at intermediate risk.

Current practice, following European guidelines, favors four-strata risk scores, at least during follow-up, because they provide clinical useful insights into the large population of patients at intermediate risk.^1^^,^^14^^,^^15^ Indeed, in the patient population, COMPERA four-strata risk score classified up to 40% of the patients with intermediate risk as having intermediate to low risk with 1-year mortality rates of < 10%. However, 5-year survival rates are compromised compared with low-risk patients, underscoring the importance of frequent monitoring and treatment adjustments in this subset of patients and the importance of treating to goal to achieve (and maintain) a low-risk profile^1^; for example, switching from phosphodiesterase type 5 inhibitors to soluble guanylate cyclase stimulators (based on the REPLACE study) or addition of prostacyclin receptor agonists (based on the Prostacycline ([PGI_2_]) Receptor Agonist In Pulmonary Arterial Hypertension, or GRIPHON, study).^29^^,^^30^ However, patients with intermediate to high risk have a 1-year mortality rate of nearly 20% and may justify more intense early treatment.

REVEAL risk scores are—in comparison with the ESC and ERS risk assessment—continuous scoring systems. When applied as continuous prediction models, the REVEAL scoring systems showed the highest statistical prognostic power and granularity in the various presented analyses. Besides this superiority from a statistical point of view, the granularity of prognostic information also may be advantageous from a clinical standpoint, even in comparison with four-strata risk scores. The impact of such more granular information on treatment and further clinical decision-making, however, will have to be delineated in further clinical trials.

The real-world data collected from patients enrolled in the GoDeep meta-registry are comparable with previous findings. For instance, the initial COMPERA 2.0 risk article indicated 79% and 50% 5-year survival rates for patients with intermediate-risk to low-risk PAH and those with intermediate-risk to high-risk PAH, respectively, which aligns with the survival rates observed in this study.^14^ Concerning published data on the prognostic power of the REVEAL 2.0 risk score, 5-year survival rates of patients with low-risk, intermediate-risk, and high-risk disease were comparable, albeit intermediate risk patients showed slightly higher mortality rates.^20^ The concordance index of the REVEAL 2.0 score was comparable with that of previously published studies (0.68 in this study vs 0.73 published previously).^20^ When comparing 5-year survival rates of patients with CTEPH at low risk, intermediate risk, and high risk with that in previous studies, the ESC and ERS 2015 showed lower mortality rates for patients with low-risk and intermediate-risk disease than in previous studies.^7^ However, this finding demands more detailed analysis in future studies, because the treatment algorithm of patients with CTEPH has changed considerably over the past years with the entrance of balloon angioplasty and combination therapies.

Our study showed that risk scores originally developed for patients with PAH (at risk for right heart failure and PH-related death) also can be used meaningfully to assign risk stratification to patients assigned to other PH groups. This contrasts with a previous study showing that the ESC and ERS risk score may not be predictive in patients with PH group 3.^9^ However, this observation was limited by a small sample size. The clinical relevance of risk scores in patients with PH assigned to group 3 will increase if more PH-centered therapies, beyond inhaled treprostinil, become available for this patient cohort.^31^ It is important to highlight that we defined several clinically significant patient groups or phenotypes and conducted subgroup analyses within these categories (eg, patients with PAH with cardiac comorbidities). Across all tested subgroups, our findings consistently demonstrated that the COMPERA four-strata risk score provides subdifferentiation of the intermediate risk group and that the REVEAL scoring systems, when used as continuous prediction models, possess the highest statistical prognostic power. Interestingly, when examining the hazard ratios directly, patients classified as having high-risk disease according to the COMPERA four-strata risk score demonstrated a hazard ratio of 11, whereas those classified as having high-risk disease using the REVEAL 2.0 and REVEAL Lite 2 scores exhibited hazard ratios of 6 and 10, respectively. Consistently, Kaplan-Meier analyses revealed that patients identified as having high-risk disease by the COMPERA four-strata risk score experienced significantly lower survival rates compared with those classified as having high-risk disease by the other risk scores. Hence, the COMPERA four-strata risk score might be particularly more effective in identifying patients with extremely high-risk disease compared with patients with the other risk scores.

Regarding PH associated with left heart disease (group 2), this study is the first to show that PAH-designed risk scores possess predictive potency. In this meta-registry analysis, patients with group 2 disease also encompass those with well-controlled heart failure with preserved ejection fraction (HFpEF)-PH demonstrating pulmonary arterial wedge pressure levels of ≤ 15 mm Hg. The classification of group 2, even in cases of borderline pulmonary arterial wedge pressure, was determined by each center, considering further clinical characteristics such as volume challenge or exercise testing results. Notably, the REVEAL scores and the four-strata risk scores showed the highest prognostic power, not only in patients with group 2 PH but also in patients with group 1 PH with cardiac comorbidities. This is commensurate with the importance of right heart function as a predictor of mortality in PH. The use of risk stratification to describe better patients recruited to studies of potential treatments for group 2 PH, and indeed to enrich these studies with patients with high-risk disease, may enable the evaluation of new therapies in this patient group.

Our study emphasizes that WHO functional class, BNP levels, and 6MWD are not exclusive to PAH, but also hold relevance in other diseases, such as left heart disease (group 2 PH) and lung diseases (group 3 PH). Although they may provide useful information in the evaluation and monitoring of patients with PAH, their interpretation also should consider the possibility of alternative underlying causes. For instance, the deterioration of left heart failure can affect these three parameters similarly to the worsening of right heart insufficiency in patients with group 2 PH or group 1 PH with cardiopulmonary comorbidities. A comprehensive evaluation of risk scores in patients with groups 2 through 5 disease thus will consider both the underlying diseases and the severity of PH and right heart failure.

Moreover, our study stands out as one of the pioneering initiatives to encompass regions of the world that often have been overlooked in previous risk assessment studies. Locations such as Johannesburg and Abu Dhabi, which traditionally were underrepresented in research assessing risk in PH, are included in our study. Notably, these regions exhibit substantial differences when compared with their Western counterparts. In Europe and America, a significant proportion of PH cases are associated with either left heart disease or chronic lung disease.^32^ However, in Africa, a distinct pattern emerges, where approximately 10% of patients received a diagnosis of PH linked to conditions such as sickle cell anemia or rheumatic heart disease.^32^ The inclusion of such diverse regions in the GoDeep meta-registry, a global PH meta-registry, allows us to consider and account for these regional disparities in PH cause and patient demographics.^21^ This approach ensures a more comprehensive and representative assessment of risk factors, acknowledging the unique characteristics and challenges faced by patients across the world.

A limitation of this study is its retrospective study design. As is often the case when relying on routinely collected clinical data, some data are missing, requiring imputation following statistical standard procedures. In mitigation, the analyses without any imputation yielded largely corresponding results. Prospective verification concerning the predictive power of the risk scores investigated in the individual PH groups is warranted. Based on the data available in the GoDeep meta-registry, only limited subgroup analyses could be undertaken, however, which again confirmed the main findings in the overall population with PH. As a further limitation, no information on interventions such as pulmonary endarterectomy or balloon pulmonary angioplasty in patients with CTEPH were included in the analysis of patients with group 4 disease. Potential biases, such as selection bias, cannot be entirely ruled out.

## Interpretation

This comprehensive study with real-world data from 15 PH centers substantially extends our understanding of the predictive power of PAH-designed risk scores, including their potential application in patients with PH beyond the PAH group.

## Funding/Support

This work is funded by the Pulmonary Vascular Research Institute and the 10.13039/100016421Cardiovascular Medical Research and Education Fund, 10.13039/100000002National Institutes of Health.

## Financial/Nonfinancial Disclosures

The authors have reported to *CHEST* the following: A. Y. has received personal fees from MSD. H. G. has received personal fees from Actelion, AstraZeneca, Bayer, BMS, GossamerBio, GSK, Janssen-Cilag, Lilly, MSD, Novartis, OMT, Pfizer, and United Therapeutics. M. R. W. reports personal fees from MorphogenIX, Janssen, Chiesi, and Aerami; grants from British Heart Foundation and NIHR; and personal fees from MSD, Benevolent AI, and Tiakis Biotech, outside the submitted work. L. H. reports personal fees and nonfinancial support from Janssen and personal fees from MSD, Gossamer, and Altavant. D. G. K. reports support for the present manuscript from the Sheffield Biomedical Research Centre and consulting fees and other payments from Jansen Pharmaceuticals, Ferrer, Altavant, MSD, and United therapeutics. P. M. H. reports personal fees from Merck Co. S. Y. C. reports personal fees from Janssen, Bayer, Pfizer, United Therapeutics, and Acceleron Pharma and is a director, officer, and shareholder of Synhale Therapeutics. S. O. reports personal fees from MSD, Janssen, and Gallenica-Ferrer. H. A. G. has received fees from Actelion, AstraZeneca, Bayer, GSK, Janssen-Cilag, Lilly, Novartis, OMT, Pfizer, and United Therapeutics. M. J. R. has received support from Janssen Pharmaceutica and Bayer Pharma AG and speaker fees from Janssen Pharmaceutica and OMT. S. S. reports personal fees from Gossamer Bio, Merck, Keros, Janssen, United Therapeutics, and Liquidia. K. T. has received personal fees from Bayer, AstraZeneca, and Gossamer. W. S. has received consultancy fees from United Therapeutics, Tiakis Biotech AG, Liquidia, Pieris Pharmaceuticals, Abivax, Pfitzer, and Medspray BV. None declared (M. Fünderich, A.L., Y. S., O. T., A. J. S., R. T. Z., P. G. W., M. Frauendorf, A. Arvanitaki, G. G., K. S., H. R. C., R. F., I. A. G., E. B., J. S. A., A. P., S. G., A. Anthi, R. W. M., J. W., F. G.)

## Supplementary Data

e-Online Data
