# Supplementary material for: Comparison of Contemporary Risk Scores in All Groups of Pulmonary Hypertension: A Pulmonary Vascular Research Institute GoDeep Meta-Registry Analysis
Source: Chest. 2024 Mar 19;166(3):585–603. doi: 10.1016/j.chest.2024.03.018 (PMC11443244; doi:10.1016/j.chest.2024.03.018)
Supplement: e-Online Data [file mmc1.docx]

## e-Table 1: Comparison of risk scores.

WHO FC = WHO functional class; 6MWd = six minute-walking distance; BNP = B-type natriuretic peptide; NT-proBNP = N-terminal pro-brain natriuretic peptide; DLCO = diffusing capacity of the lung for carbon monoxide; RAP = right atrial pressure; PVR = pulmonary vascular resistance; VO2 peak = peak oxygen consumption; VE/VCO2 = minute ventilation/carbon dioxide production; RA area = right atrial area; SvO2 = mixed venous oxygen saturation; TAPSE = tricuspid annular plane systolic excursion; sPAP = systolic pulmonary arterial pressure; SVI = stroke volume index; cMRI = cardiac magnetic resonance imaging; *italic = not included in calculation in this study*.

| **Parameter** | **REVEAL 2.0** | | | **REVEAL Lite 2** | **ESC/ERS 2022** | **COMPERA 3-strata** | **COMPERA 4-strata** |
| --- | --- | --- | --- | --- | --- | --- | --- |
| WHO Group 1 Subgroup | | X |  | |  |  |  |
| Demographics | | X |  | |  |  |  |
| Renal function | | X | X | |  |  |  |
| WHO FC | | X | X | | X | X | X |
| Vital Signs | | X | X | |  |  |  |
| 6MWd | | X | X | | X | X | X |
| BNP/NT-proBNP | | X | X | | X | X | X |
| Pericardial effusion | | X |  | | X |  |  |
| DLCO | | X |  | |  |  |  |
| RAP | | X |  | | X |  |  |
| PVR | | X |  | |  |  |  |
| VO2 peak | |  |  | | X |  |  |
| VE/VCO2 slope | |  |  | | X |  |  |
| RA area | |  |  | | X |  |  |
| Cardiac index | |  |  | | X |  |  |
| *SvO_2_* | |  |  | | *X* |  |  |
| TAPSE/sPAP ratio | |  |  | | *X* |  |  |
| SVI | |  |  | | *X* |  |  |
| *Hospitalization within 6 months* | | *X* |  | |  |  |  |
| *Clinical observations* | |  |  | | *X* |  |  |
| *cMRI* | |  |  | | *X* |  |  |

## e-Figure 1: Imputation reliability.

The figure shows the hazard ratios obtained from the unimputed data set plotted against the hazard ratios from the imputed data set with a missingness of at most 30% and 40%. The following procedure was used for imputation: Patients were included if they had at least two of the parameters WHO functional class, 6MWD and BNP. Two different imputations were conducted: In the first imputation, missing data was allowed up to 30%. This resulted in 7440 patients and several parameters being imputed. In the second imputation, up to 40% missing data was allowed, and no additional patients were excluded. This imputation involved 8565 patients and multiple parameters. Continuous variables were log-transformed before imputation, and the analysis was conducted on both datasets. Hazard ratios from the imputed datasets were compared to those from the dataset without any imputation. The mean standard deviation for the imputation with a maximum of 30% missing data was 0.16 compared to the data without imputation, and 0.15 for the imputation with a maximum of 40%. Both imputations showed that hazard ratios were not overestimated, and the results were presented using the larger dataset in the main analysis.

HR = hazard ratio; 6MWd = six minute-walking distance; BNP = B-type natriuretic peptide.


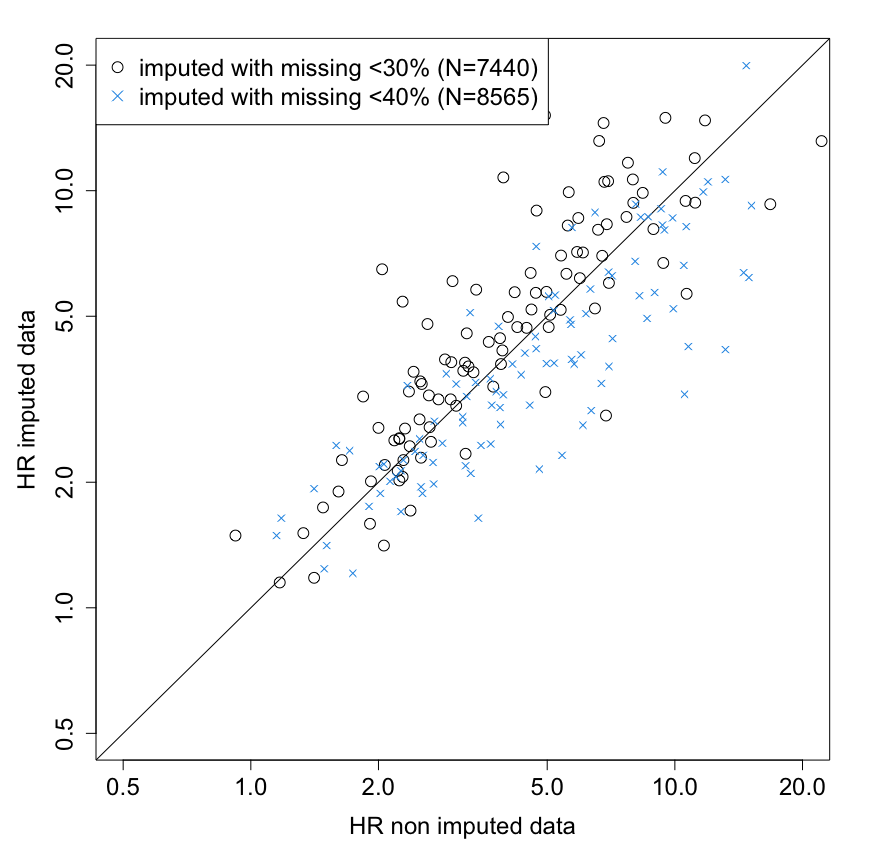


e-Table 2: Baseline characteristics of the non-imputed study population stratified by PH group 1-4

PH patients’ characteristics at baseline and stratified by PH Group. Only patients with available 6MWD, BNP, and WHO FC were included. Median and interquartile range are given.

PH = pulmonary hypertension; WHO FC = WHO functional class; 6MWd = six minute-walking distance; BNP = B-type natriuretic peptide; CI = cardiac index, mPAP = mean pulmonary arterial pressure, PAWP = pulmonary arterial wedge pressure, PVR = pulmonary vascular resistance.

e-Table 3: Comorbidities of the non-imputed study population stratified by PH group 1-4

PH patients’ comorbidiites at baseline and stratified by PH Group. Only patients with available 6MWD, BNP, and WHO FC were included. Median and interquartile range are given.

PH = pulmonary hypertension.

e-Table 4: Risk score classification of the non-imputed study population stratified by PH group 1-4

Risk scores classification at baseline and stratified by PH Group. Only patients with available 6MWD, BNP, and WHO FC were included.

PH = pulmonary hypertension; WHO FC = WHO functional class; 6MWd = six minute-walking distance; BNP = B-type natriuretic peptide; int. = intermediate; int.-high = intermediate-high; int.-low = intermediate-low.

e-Figure 2: Kaplan-Meier curves of risk scores (non-imputed study population).

Survival of all PH patients stratified by risk score. Kaplan-Meier curves with 95 % confidence bands.

**a)** Kaplan-Meier curve stratified by REVEAL 2.0 risk score.
**b)** Kaplan-Meier curve stratified by REVEAL Lite 2 risk score.
**c)** Kaplan-Meier curve stratified by ESC/ERS 2022 risk score.
**d)** Kaplan-Meier curve stratified by COMPERA 3-strata risk score.
**e)** Kaplan-Meier curve stratified by COMPERA 4-strata risk score.

PH = pulmonary hypertension; int. = intermediate; int.-high = intermediate-high; int.-low = intermediate-low.
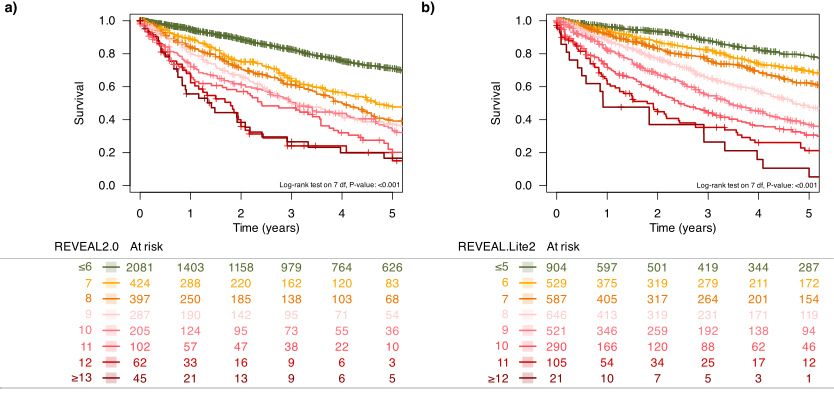

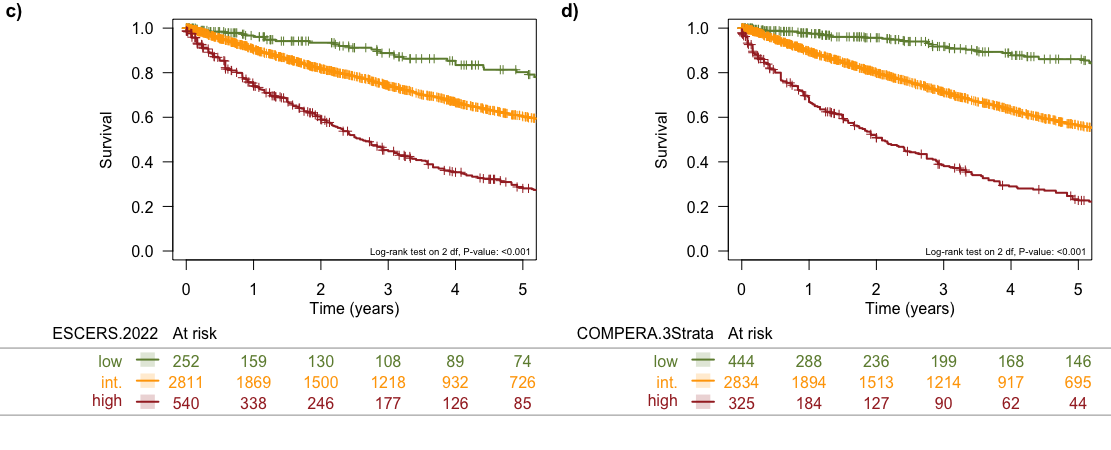

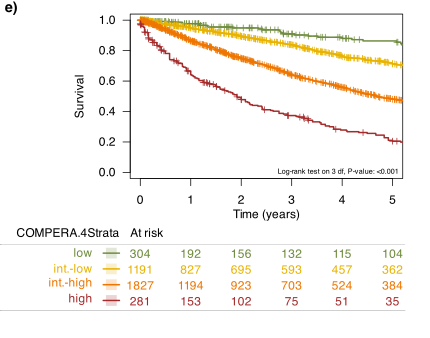


e-Table 5: Predictive power of all included risk scores, non-imputed study population.

C-Index and the difference of AIC estimates between the ESC/ERS 2022 score and the respective risk score of the Cox proportional hazards model based on non-imputed data are shown. Center and diagnosis decade are included as stratification variables, as is center included as cluster. Table with values for risk scores at baseline for overall PH and groups 1-4.

∆AIC = difference of akaike information criterion between the ESC/ERS 2022 score and the respective risk score; C-Index = concordance index; PH = pulmonary hypertension.

*p-values <0.001 in comparison to ESC/ERS 2022 score.

|  |  | **Reveal 2.0** | **Reveal Lite 2** | **ESC/ERS 2022** | **COMPERA 3-strata** | **COMPERA**  **4-strata** |
| --- | --- | --- | --- | --- | --- | --- |
| PH overall | AIC | 178 | 179 | 0 | 41 | 129 |
|  | C-Index | 0.67* | 0.67* | 0.60 | 0.60 | 0.64* |
|  |  |  |  |  |  |  |
| Group 1 | AIC | 88 | 70 | 0 | 6 | 47 |
|  | C-Index | 0.70* | 0.69* | 0.62 | 0.61 | 0.67* |
|  |  |  |  |  |  |  |
| Group 2 | AIC | -2 | -4 | 0 | -7 | -2 |
|  | C-Index | 0.65 | 0.61 | 0.60 | 0.58 | 0.58 |
|  |  |  |  |  |  |  |
| Group 3 | AIC | 9 | 22 | 0 | 0 | 14 |
|  | C-Index | 0.67 | 0.67* | 0.60 | 0.59 | 0.64 |
|  |  |  |  |  |  |  |
| Group 4 | AIC | 29 | 13 | 0 | 0 | 3 |
|  | C-Index | 0.73* | 0.69* | 0.62 | 0.61 | 0.64 |
|  |  |  |  |  |  |  |

e-Figure 3: Forest plots with Hazard Ratios (all in relation to the lowest risk category of the respective risk score), non-imputed study population.

**a)** Forest plot for the overall PH group stratified by risk score
**b)** Forest plot for PH group 1 stratified by risk score.
**c)** Forest plot for PH group 2 stratified by risk score.
**d)** Forest plot for PH group 3 stratified by risk score.
**e)** Forest plot for PH group 4 stratified by risk score.

PH = pulmonary hypertension; HR = hazard ratio; lwr.CI = lower 95% confidence bound; upr.CI = upper 95% confidence bound; int. = intermediate; int.-high = intermediate-high; int.-low = intermediate-low.

a)


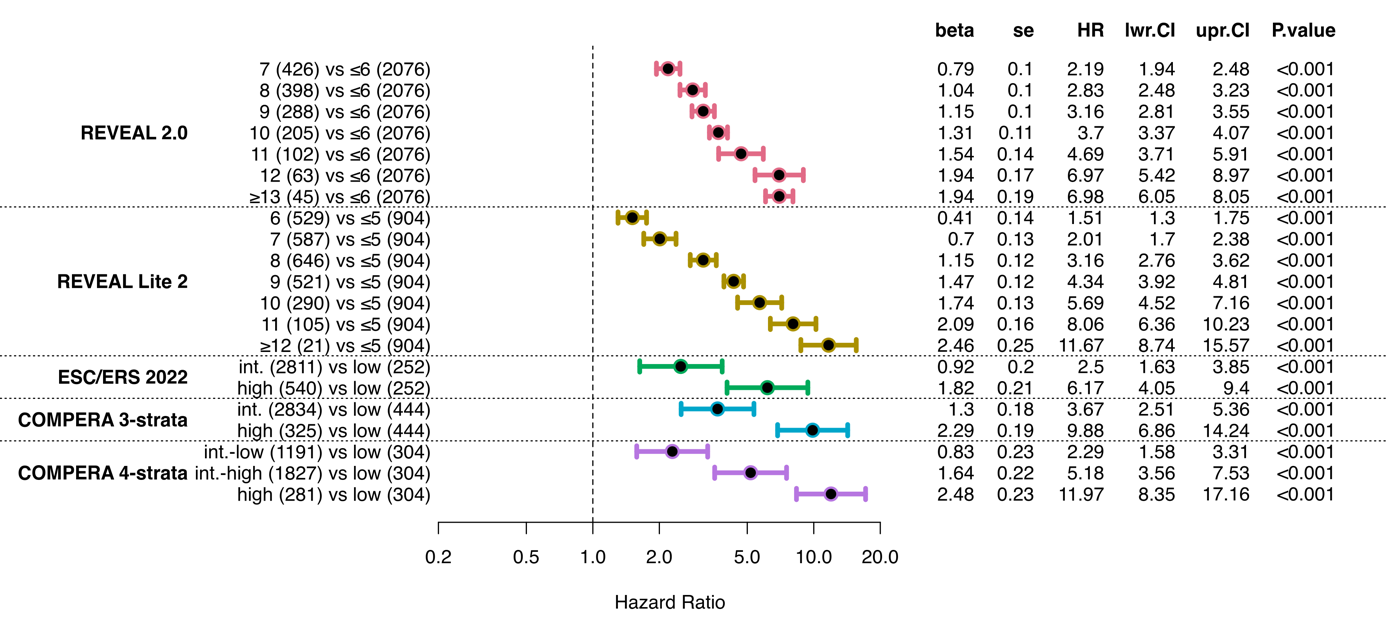


b)


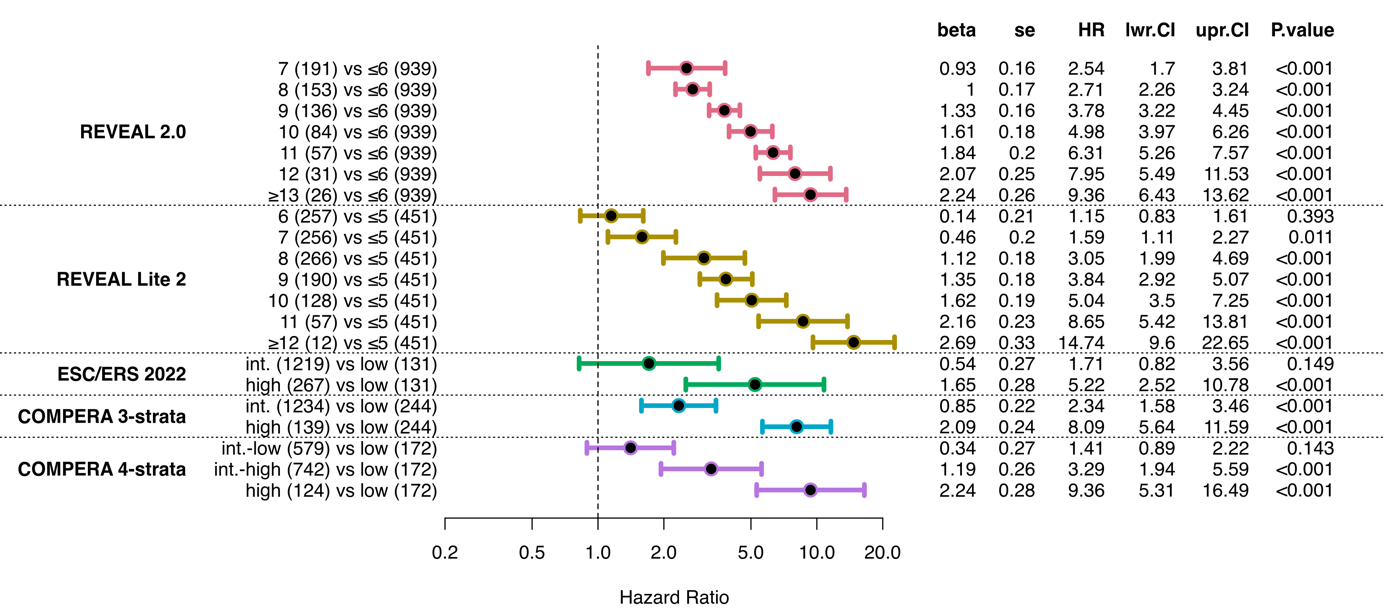
c)


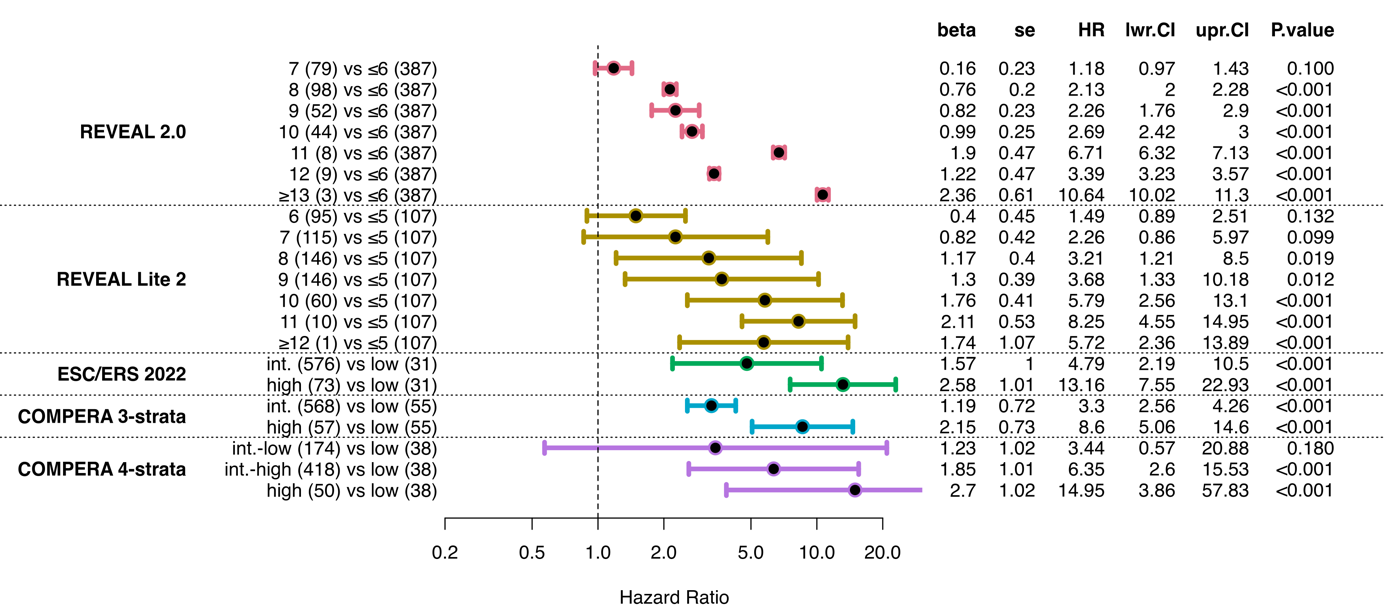
d)


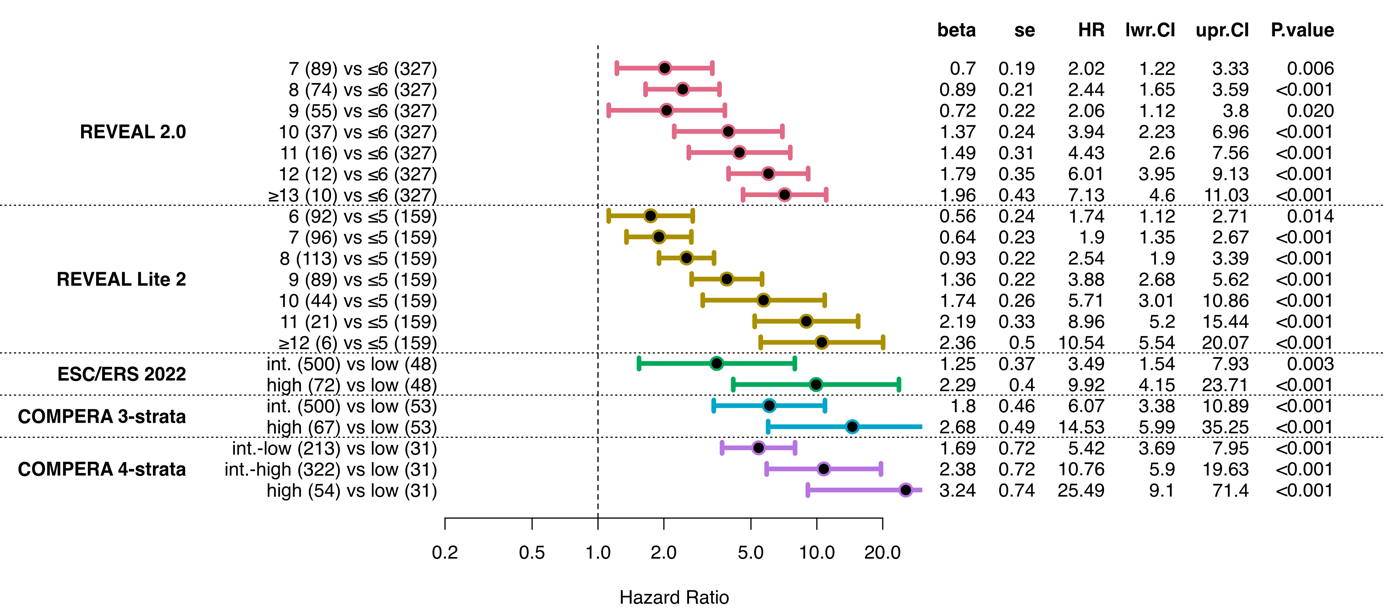


e)


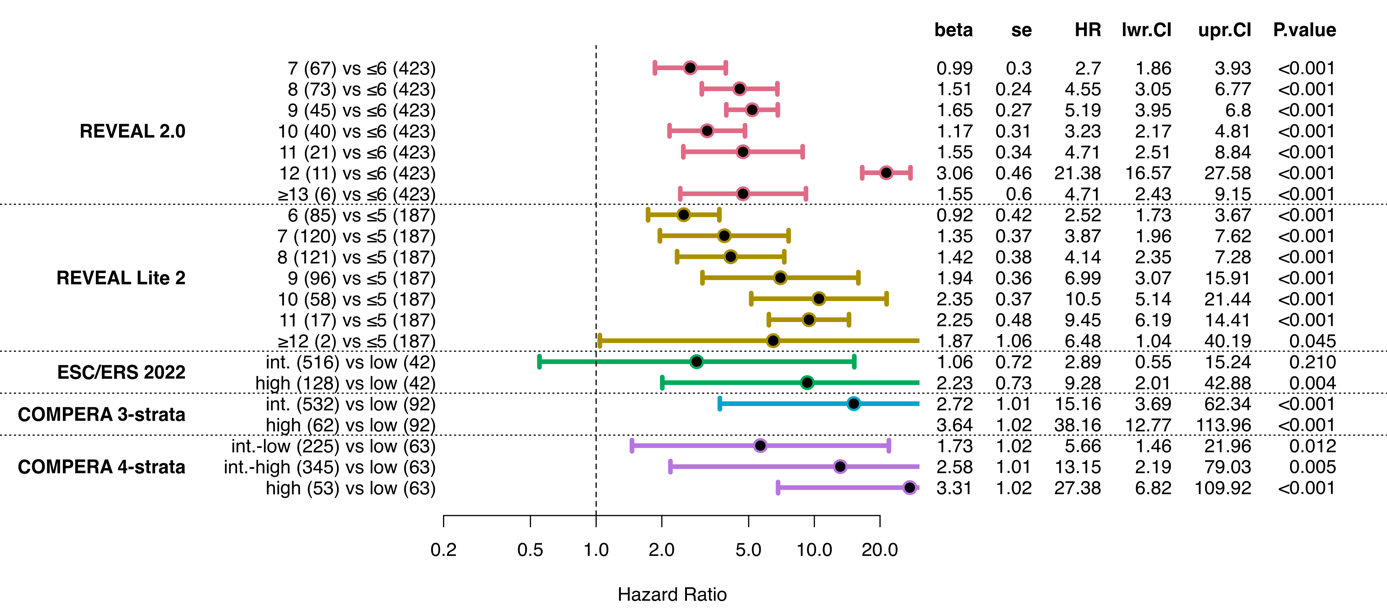


e-Figure 4: Kaplan-Meier curves of 3- and 4-strata risk scores for PH group 1 – 4, non-imputed study population.

Survival rates for each PH group 1 - 4 stratified by REVEAL 2.0, REVEAL Lite 2, ESC/ERS 2022, COMPERA 3-strata and COMPERA 4-strata, respectively. Kaplan-Meier curves with 95 % confidence bands are shown for patients in each risk score group.
**a)** Survival rates for REVEAL 2.0.
**b)** Survival rates for REVEAL Lite 2.
**c)** Survival rates for ESC/ERS 2022.
**d)** Survival rates for COMPERA 3-strata.
**e)** Survival rates for COMPERA 4-strata.
PH = pulmonary hypertension; int. = intermediate; int.-high = intermediate-high; int.-low = intermediate-low.

**
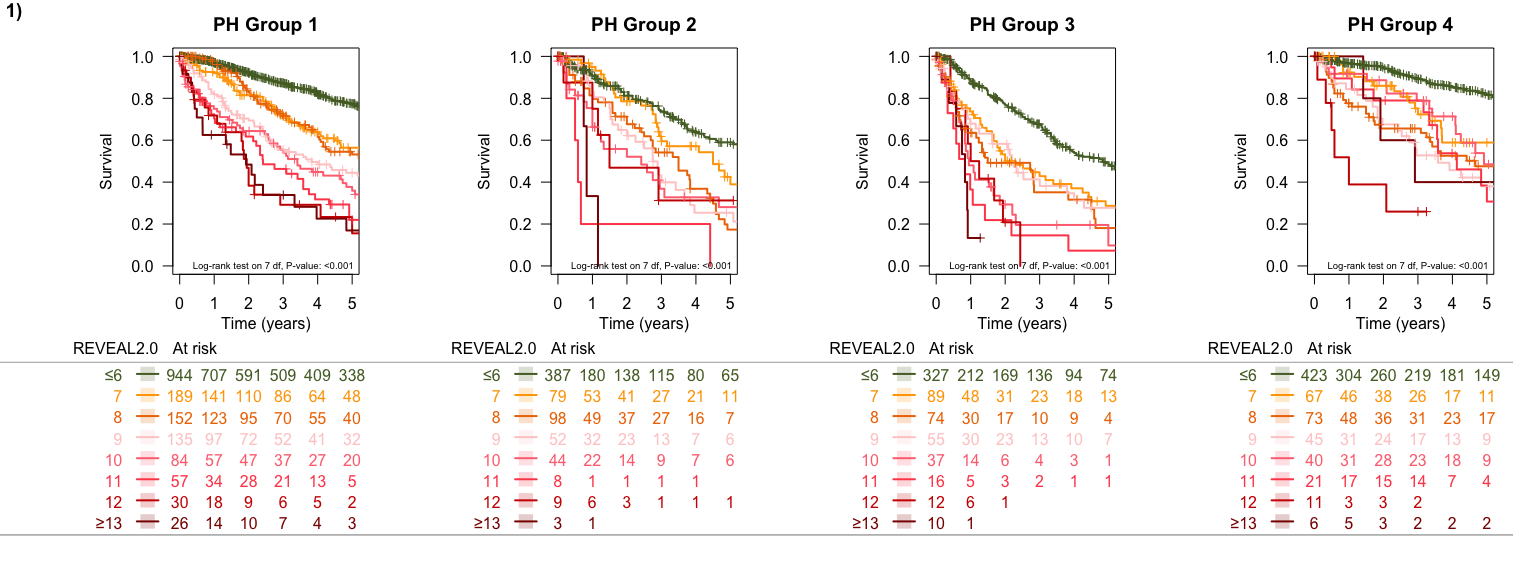

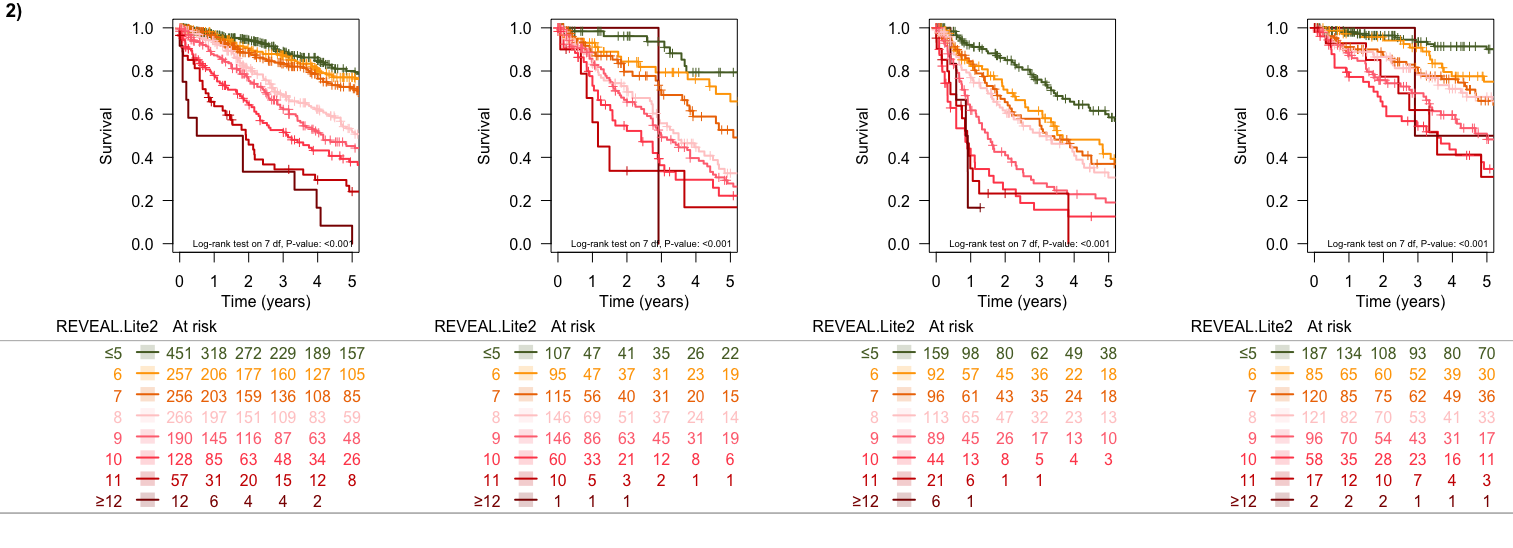

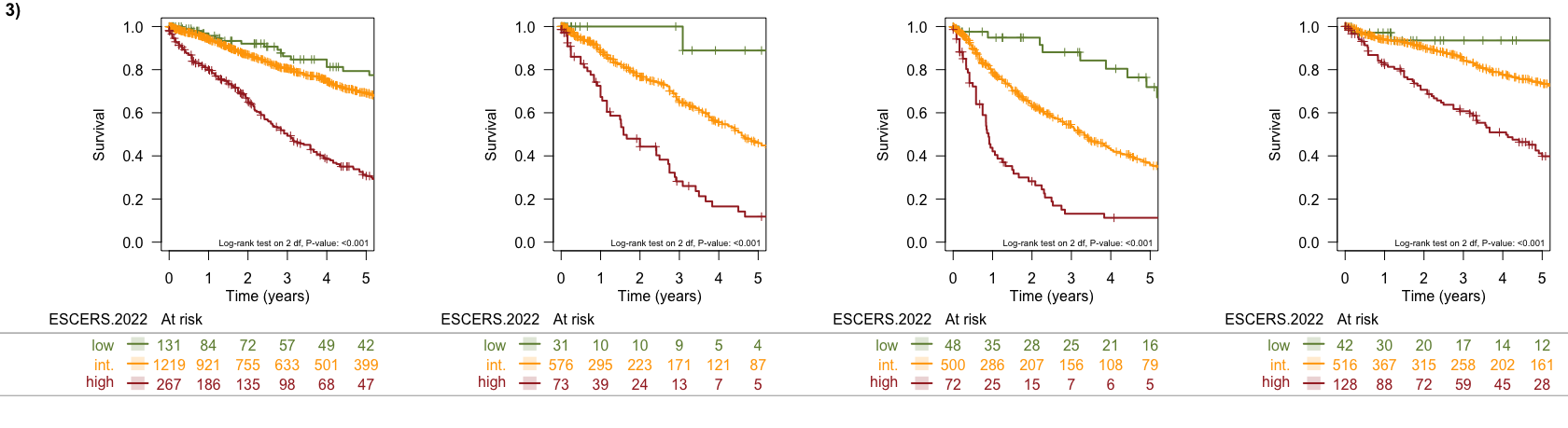

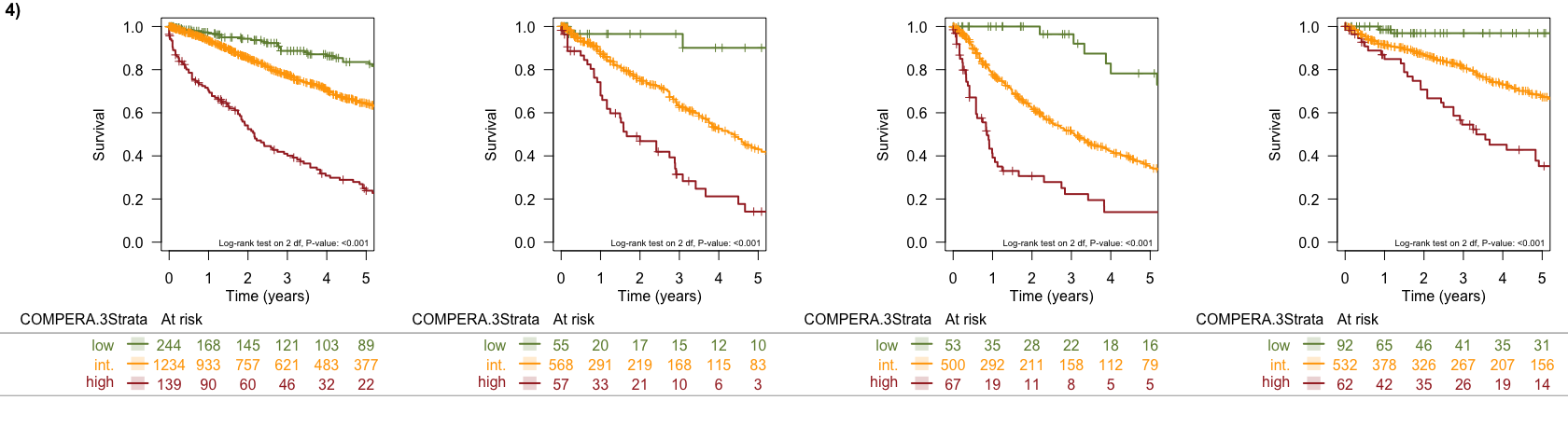

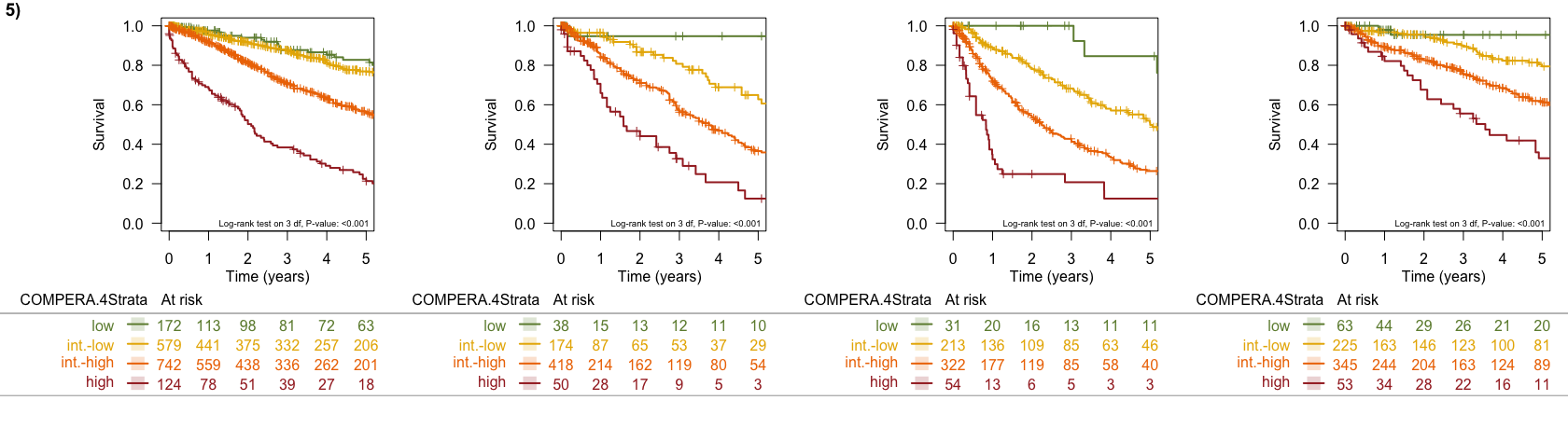
**

## e-Figure 5: Forest plot of COMPERA 4-strata risk score with Hazard Ratios in relation to the intermediate-low risk category, non-imputed study population.

PH = pulmonary hypertension; HR = hazard ratio, lwr.CI = lower 95% confidence bound; upr.CI = upper 95% confidence bound; int.-high = intermediate-high; int.-low = intermediate-low.


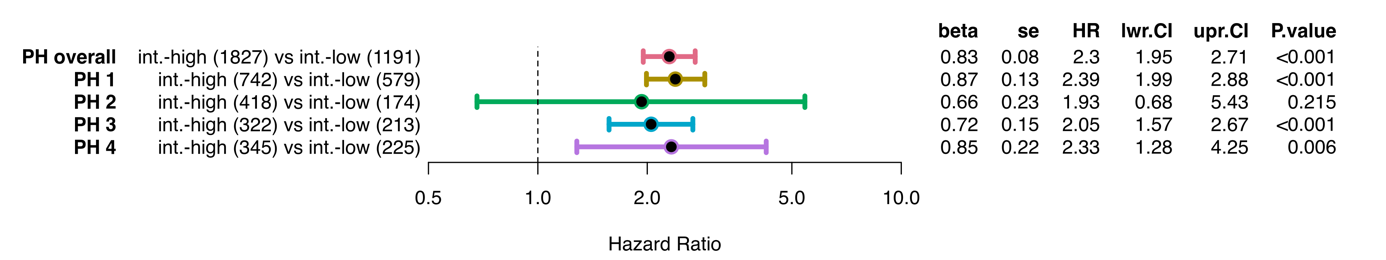


e-Table 6: Predictive power for subgroups analyses of all included risk scores, imputed study population.

C-Index and the difference of AIC estimates between the ESC/ERS 2022 score and the respective risk score of the Cox proportional hazards model based on non-imputed data are shown. Center and diagnosis decade are included as stratification variables, as is center included as cluster. Table with values for risk scores at baseline for overall PH and groups 1-4 for patients with PVR ≥5 WU and PVR <5 WU and for

**a)** Patients with PVR <5 WU. **b)** Patients PVR ≥5 WU **c)** Further subgroup analyses:
(i) Group 1.1: Idiopathic pulmonary arterial hypertension (IPAH)
(ii) Group 1.4.1: Connective tissue disease-associated pulmonary arterial hypertension
(iii) Group 1.4.4: Congenital heart disease-associated pulmonary arterial hypertension
(iv) Group 2 PH patients with isolated postcapillary PH (i.e., PVR ≤2 WU)
(v) Group 2 PH patients with combined pre- and postcapillary PH (i.e., PVR >2 WU)
(vi) Group 3.1: PH associated with obstructive lung disease
(vii) Group 3.2: PH associated with restrictive lung disease
(viii) PAH patients with cardiac comorbidities (defined as the presence of at least three of the following comorbidities: arterial hypertension, obesity, diabetes, coronary heart disease, and atrial fibrillation)

∆AIC = difference of akaike information criterion between the ESC/ERS 2022 score and the respective risk score; C-Index = concordance index; PH = pulmonary hypertension; IPAH = idiopathic pulmonary arterial hypertension; CTDPH = connective tissue disease-associated pulmonary hypertension; CHDPH = congenital heart disease-associated pulmonary hypertension; IpcPH = isolated post-capillary pulmonary hypertension; CpcPH = combined post- and pre-capillary pulmonary hypertension; COPD = chronic obstructive pulmonary disease; ILD = interstitial lung disease.

*p-values <0.001 in comparison to ESC/ERS 2022 score.

**a)**

|  |  | **Reveal 2.0** | **Reveal**  **Lite 2** | **ESC/ERS 2022** | **COMPERA 3-strata** | **COMPERA**  **4-strata** |
| --- | --- | --- | --- | --- | --- | --- |
| PH overall | **∆**AIC | 136 | 99 | 0 | 44 | 71 |
|  | C-Index | 0.66* | 0.65* | 0.57 | 0.59* | 0.62* |
| Group 1 | **∆**AIC | 72 | 64 | 0 | 17 | 35 |
|  | C-Index | 0.71* | 0.71* | 0.60 | 0.62 | 0.66 |
| Group 2 | **∆**AIC | -8 | 4 | 0 | -7 | -4 |
|  | C-Index | 0.61 | 0.62* | 0.57 | 0.56 | 0.58 |
| Group 3 | **∆**AIC | 22 | 4 | 0 | 3 | 5 |
|  | C-Index | 0.66* | 0.64* | 0.56 | 0.58 | 0.61* |
| Group 4 | **∆**AIC | 1 | -9 | 0 | 5 | 3 |
|  | C-Index | 0.65 | 0.61* | 0.56 | 0.58 | 0.57 |

**b)**

|  |  | **Reveal 2.0** | **Reveal**  **Lite 2** | **ESC/ERS 2022** | **COMPERA 3-strata** | **COMPERA**  **4-strata** |
| --- | --- | --- | --- | --- | --- | --- |
| PH overall | **∆**AIC | 262 | 248 | 0 | 83 | 216 |
|  | C-Index | 0.65* | 0.65* | 0.57 | 0.58 | 0.63* |
| Group 1 | **∆**AIC | 134 | 138 | 0 | 18 | 101 |
|  | C-Index | 0.68* | 0.68* | 0.58 | 0.59 | 0.65* |
| Group 2 | **∆**AIC | 45 | 12 | 0 | 16 | 21 |
|  | C-Index | 0.64 | 0.60 | 0.54 | 0.58 | 0.59 |
| Group 3 | **∆**AIC | 22 | 41 | 0 | -2 | 16 |
|  | C-Index | 0.62* | 0.63* | 0.57 | 0.55 | 0.59 |
| Group 4 | **∆**AIC | 16 | 17 | 0 | 13 | 16 |
|  | C-Index | 0.65* | 0.67* | 0.59 | 0.59 | 0.63 |

**c)**

|  |  | **Reveal 2.0** | **Reveal**  **Lite 2** | **ESC/ERS 2022** | **COMPERA 3-strata** | **COMPERA**  **4-strata** |
| --- | --- | --- | --- | --- | --- | --- |
| IPAH | **∆**AIC | 117 | 98 | 0 | 40 | 84 |
|  | C-Index | 0.68* | 0.67* | 0.57 | 0.60 | 0.65* |
| CTDPH | **∆**AIC | 54 | 40 | 0 | -14 | 31 |
|  | C-Index | 0.67* | 0.67* | 0.57 | 0.56 | 0.64* |
| CHDPH | **∆**AIC | 27 | 31 | 0 | 12 | 23 |
|  | C-Index | 0.66 | 0.73* | 0.54 | 0.60 | 0.67 |
| IpcPH | **∆**AIC | 9 | -1 | 0 | 5 | 4 |
|  | C-Index | 0.62 | 0.55 | 0.55 | 0.58 | 0.55 |
| CpcPH | **∆**AIC | 17 | 7 | 0 | -6 | 5 |
|  | C-Index | 0.62 | 0.59 | 0.55 | 0.53 | 0.57 |
| PH-COPD | **∆**AIC | 32 | 21 | 0 | 10 | 29 |
|  | C-Index | 0.60* | 0.60* | 0.54 | 0.55 | 0.59* |
| PH-ILD | **∆**AIC | 16 | 12 | 0 | -1 | 3 |
|  | C-Index | 0.63 | 0.63 | 0.58 | 0.56 | 0.60 |
| PAH patients with cardiac comorbidities | **∆**AIC | 14 | 22 | 0 | 3 | 7 |
|  | C-Index | 0.70* | 0.70* | 0.57 | 0.58 | 0.62 |
|  |  |  |  |  |  |  |
